# Blood pressure and risk of venous thromboembolism: a cohort analysis of 5.5 million UK adults and Mendelian randomization studies

**DOI:** 10.1093/cvr/cvac135

**Published:** 2022-08-29

**Authors:** Milad Nazarzadeh, Zeinab Bidel, Hamid Mohseni, Dexter Canoy, Ana-Catarina Pinho-Gomes, Abdelaali Hassaine, Abbas Dehghan, David-Alexandre Tregouet, Nicholas L Smith, Kazem Rahimi, INVENT Consortium

**Affiliations:** Deep Medicine, Oxford Martin School, University of Oxford, 1st Floor, Hayes House, 75 George Street, Oxford OX1 2BQ, UK; Medical Science Division, Nuffield Department of Women’s and Reproductive Health, University of Oxford, UK; Deep Medicine, Oxford Martin School, University of Oxford, 1st Floor, Hayes House, 75 George Street, Oxford OX1 2BQ, UK; Medical Science Division, Nuffield Department of Women’s and Reproductive Health, University of Oxford, UK; Deep Medicine, Oxford Martin School, University of Oxford, 1st Floor, Hayes House, 75 George Street, Oxford OX1 2BQ, UK; Deep Medicine, Oxford Martin School, University of Oxford, 1st Floor, Hayes House, 75 George Street, Oxford OX1 2BQ, UK; Medical Science Division, Nuffield Department of Women’s and Reproductive Health, University of Oxford, UK; Faculty of Medicine, University of New South Wales, Sydney, Australia; NIHR Oxford Biomedical Research Centre, Oxford, UK; Medical Science Division, Nuffield Department of Women’s and Reproductive Health, University of Oxford, UK; Deep Medicine, Oxford Martin School, University of Oxford, 1st Floor, Hayes House, 75 George Street, Oxford OX1 2BQ, UK; Medical Science Division, Nuffield Department of Women’s and Reproductive Health, University of Oxford, UK; Department of Biostatistics and Epidemiology, School of Public Health, Imperial College London, UK; INSERM UMR_S 1219, Bordeaux Population Health Research Center, University of Bordeaux, Bordeaux, France; Department of Epidemiology, University of Washington, Seattle, WA; Kaiser Permanente Washington Health Research Institute, Kaiser Permanente Washington, Seattle, WA; Department of Veterans Affairs Office of Research and Development, Seattle Epidemiologic Research and Information Center, Seattle, WA; Deep Medicine, Oxford Martin School, University of Oxford, 1st Floor, Hayes House, 75 George Street, Oxford OX1 2BQ, UK; Medical Science Division, Nuffield Department of Women’s and Reproductive Health, University of Oxford, UK; NIHR Oxford Biomedical Research Centre, Oxford, UK

**Keywords:** Systolic blood pressure, Venous thromboembolism, Mendelian randomization, Pulmonary embolism, Deep venous thrombosis

## Abstract

**Aims:**

Evidence for the effect of elevated blood pressure (BP) on the risk of venous thromboembolism (VTE) has been conflicting. We sought to assess the association between systolic BP and the risk of VTE.

**Methods and results:**

Three complementary studies comprising an observational cohort analysis, a one-sample and two-sample Mendelian randomization were conducted using data from 5 588 280 patients registered in the Clinical Practice Research Datalink (CPRD) dataset and 432 173 UK Biobank participants with valid genetic data. Summary statistics of International Network on Venous Thrombosis genome-wide association meta-analysis was used for two-sample Mendelian randomization. The primary outcome was the first occurrence of VTE event, identified from hospital discharge reports, death registers, and/or primary care records. In the CPRD cohort, 104 017(1.9%) patients had a first diagnosis of VTE during the 9.6-year follow-up. Each 20 mmHg increase in systolic BP was associated with a 7% lower risk of VTE [hazard ratio: 0.93, 95% confidence interval (CI): (0.92–0.94)]. Statistically significant interactions were found for sex and body mass index, but not for age and subtype of VTE (pulmonary embolism and deep venous thrombosis). Mendelian randomization studies provided strong evidence for the association between systolic BP and VTE, both in the one-sample [odds ratio (OR): 0.69, (95% CI: 0.57–0.83)] and two-sample analyses [OR: 0.80, 95% CI: (0.70–0.92)].

**Conclusion:**

We found an increased risk of VTE with lower BP, and this association was independently confirmed in two Mendelian randomization analyses. The benefits of BP reduction are likely to outweigh the harms in most patient groups, but in people with predisposing factors for VTE, further BP reduction should be made cautiously.

**Time for primary review: 165 days**

## 1. Introduction

Venous thromboembolism (VTE), which comprises deep vein thrombosis and pulmonary embolism (DVT/PE), is a paramount public health concern, with an estimated annual incidence rate of 100–180 per 100 000 person-years in non-hospitalized patients in Europe and the United States.^1,2^ VTE is mostly a disease of old age, with a marked increase in incidence after the age of 65, and it is also more common in men than in women.^3,4^ Besides the mortality associated with PE, which is a common cause of sudden death, the long-term complications of VTE, including post-thrombotic syndrome and pulmonary hypertension, are a significant cause of morbidity.^5^ The high incidence of VTE together with the high costs associated with treatment and complications cause a high burden to healthcare systems, further exacerbated by population ageing.^6,7^ Although risk factors for VTE in acute settings are well established,^8^ current understanding of modifiable risk factors for VTE in patients in the community is limited^9^, and apart from body mass index (BMI)^10–12^ and cholesterol-lowering with statins,^13^ the role of traditional cardiovascular risk factors remains unclear. Several observational studies have investigated associations between blood pressure (BP) and the risk of VTE. A tabular meta-analysis of ten studies, including 42 555 participants, showed that hypertension was associated with an ∼50% higher risk of VTE in both case-control and cohort studies.^14^ In contrast, an individual participant data (IPD) meta-analysis of 244 865 individuals from nine prospective cohort studies showed that higher systolic BP was associated with a lower risk of VTE.^15^ A subsequent observational analysis of two separate cohorts with 3362 events found inconsistent associations between them and could not resolve the issue.^16^ Considering the conflicting evidence currently available from observational studies, we sought to investigate the observational relationship between systolic BP and incident VTE using large-scale population-based healthcare data and to assess the causal nature of association using genetic data.

## 2. Methods

### 2.1 Observational cohort analysis: Clinical Practice Research Datalink

This analysis was conducted using linked electronic health records from the UK Clinical Practice Research Datalink (CPRD) study (www.cprd.com) from its inception on 1 January 1985 to 31 December 2015. A total of 6 613 644 patients, aged 30–90 years, and with at least one BP measurement were included in the study. Patients entered the cohort at the date of the earliest BP measurement if they had at least 1 year of follow-up. They were followed up until the earliest occurrence of a VTE event, or death, or end of registration with the general practice, or 31 December 2015. We excluded patients if they had any of the following: (i) previously documented myocardial infarction, ischaemic heart disease, stroke, transient ischaemic attack, heart failure, chronic kidney disease, peripheral arterial disease, atrial fibrillation, cancer, or VTE; (ii) past or current prescription of lipid-lowering or anti-hypertensive medications.

Study exposure was systolic BP per 20 mmHg increase (consistent with other major BP studies),^17–19^ and the primary outcome was the first occurrence of a VTE event, identified from hospital discharge reports, death registers, and/or primary care records. VTE was defined using an externally validated algorithm^20^ with the International Classification of Diseases (ICD) diagnostic codes described in Supplementary material online, *Table S1*. Cox proportional hazard models were used to estimate the multivariable-adjusted hazard ratio (HR) for VTE. A generalized estimating equation model was used to estimate the adjusted regression coefficient corrected for regression dilution to account for measurement error and short-term variations in systolic BP during follow-up (mean of 6.7 BP measurements per patient).^21^ Missing data were addressed using multiple imputations by expectation-maximization with bootstrapping, generating five imputations.^22^ Several sensitivity analyses were performed to test the robustness of the findings. The details of methods and sensitivity analyses have been described in Supplementary material online, *Method S1*. The Trent Multi-Centre Research Ethics Committee (05/MRE/04/87) has approved the use of anonymized CPRD data.

### 2.2 Mendelian randomization studies

#### 2.2.1 One-sample Mendelian randomization

We used the UK Biobank data, which is a large prospective cohort study that included 502 602 participants aged 40–69 years, recruited between 2006 and 2010 from 22 assessment centres across the UK. Details of the UK Biobank design have been published elsewhere.^23,24^ UK Biobank genotype data were imputed with IMPUTE4 using the Haplotype Reference Consortium and the UK10K + 1000 Genomes panel^25^ to identify ∼96 million variants for 487 381 participants. We excluded 55 208 individuals who were not white British, had a variant call rate <98%, and were outliers based on heterozygosity. Finally, we included 432 173 participants in the Mendelian randomization study. We built a weighted polygenic risk score as an instrumental variable for systolic BP using independent genetic variants (linkage disequilibrium r^2^ < 0.05) with minor allele frequency > 0.01 and *P* < 5 × 10^−8^ at a genome-wide level. Overall, 276 genetic variants were selected, all with imputation quality > 0.9 that have been shown to be associated with systolic BP in a genome-wide association (GWAS) meta-analysis including over one million participants of European ancestry (see Supplementary material online, *Table S2*).^26^ The details of constructing polygenic risk scores from GWAS results have been described in Supplementary material online, *Method S2*. The outcome was defined as VTE episode, including PE and DVT. VTE cases were extracted based on hospital discharge reports and death registers, that were linked to the UK Biobank using the same ICD codes described for the CPRD cohort analysis (see Supplementary material online, *Table S1*).

The two-stage least-squares approach was employed in a one-sample setting using UK Biobank individual-level data. In the first stage, we regressed the measured systolic BP on the weighted polygenic risk score as an instrumental variable through a linear regression model. In the second stage, the binary outcome regressed on fitted values derived from the first stage. For the second stage, we used binary logistic regression adjusted for age, sex, UK biobank assessment centre, genetic batch, population stratification (the first ten genetic principal components), and up to third-degree relatedness based on kinship coefficients (> 0.044).

As there is strong evidence from clinical trials about the effect of systolic BP on coronary heart disease and stroke,^27^ we conducted a positive control analysis to test the validity of the instrumental variable. Besides, an unweighted polygenic risk score was used to check the robustness of the weighting approach. Finally, to check the possible effect of confounder variables, we further adjusted the model for BMI, alcohol intake frequency, smoking status, total cholesterol, low-density lipoprotein (LDL), high-density lipoprotein (HDL), BP-lowering medications use, and cardiovascular comorbidities. We also restricted the analysis to people who were not related to any other participants to see if relatedness had an impact on the main conclusion.

#### 2.2.2 Two-sample Mendelian randomization

We performed two-sample Mendelian randomization, which uses summary statistics estimated in two non-overlapping data.^28^ Compared with one-sample Mendelian randomization, the two-sample method overcomes weak instrument bias, which is a limitation of one-sample Mendelian randomization that can lead to biases towards the confounded observational analysis.^29^ Also, the statistical power of two-sample Mendelian randomization tends to be higher because it combines summary results from GWAS consortia.^29^

In this analysis, we used 276 genetic variants for systolic BP as described in the one-sample Mendelian randomization section but the estimations for outcome were derived from the International Network Against Venous Thrombosis (INVENT) Consortium.^30^ Before doing the statistical analysis, the summary estimations of genetic variants were harmonized.^28,31^ The random-effect inverse-variance weighted technique was employed as the primary method, assuming that either all of the instruments are valid or that any horizontal pleiotropy is balanced.^32^ As sensitivity analyses, we used multiple Mendelian randomization methods with varied assumptions to assess the robustness and reliability of our findings. We employed the weighted median method,^33^ which is consistent if at least 50% of the weight comes from valid instrumental variables.^34^ The Mendelian Randomization Pleiotropy RESidual Sum and Outlier method was used to test and, if needed, to correct for any possible horizontal pleiotropic outliers in the analysis.^35^ The MR-Egger regression method was used to assess the presence of pleiotropy.^36^

We examined the heterogeneity of the estimates by using a scatter plot and applying Cochran’s Q test.^37^ We also assessed the probable directional pleiotropy using a funnel plot similar to that being used to determine publication bias in meta-analysis.^37^

Statistical analyses were performed using Stata Statistical Software, release 14 (StataCorp LP) and R, version 3.3 (R Foundation for Statistical Computing, Vienna, Austria). The ‘MendelianRandomization’ and ‘TwoSampleMR’ packages for R were used to implement the Mendelian randomization analyses.^38,39^ The study protocol was approved by the UK Biobank scientific committee (project number 42447). UK Biobank study obtained informed consent from the study participants and approval from its institutional review board. This study conforms to the principles outlined in the Declaration of Helsinki.

## 3. Results

### 3.1 CPRD observational findings

**Figure 1:**
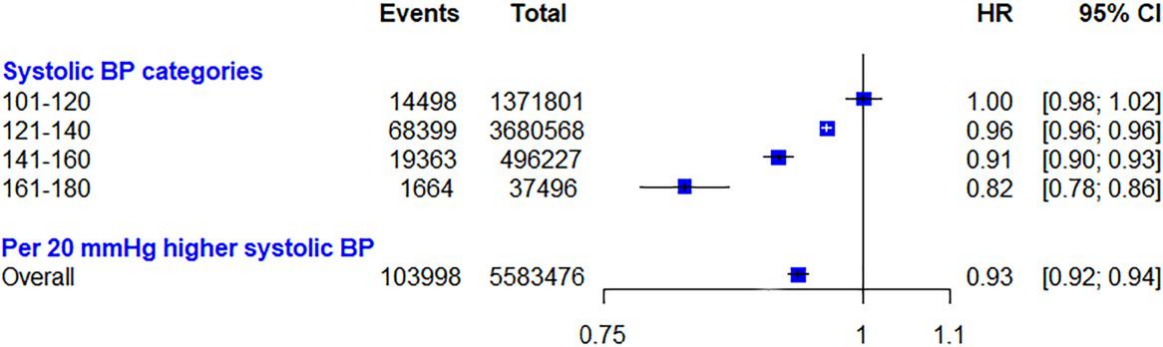
The risk of venous thromboembolism (VTE) by categories of systolic blood pressure (BP) in the CPRD cohort analysis. Hazard ratio (HR) and 95% confidence interval (CI) are displayed using floating absolute risks and corrected for regression dilution. Models are adjusted for age, sex, body mass index, smoking, alcohol, total cholesterol, LDL cholesterol, HDL cholesterol, practice level, and anticoagulant prescription during follow-up. We used ICD diagnosis codes for the identification of outcomes. Given that a patient might experience both pulmonary embolism and deep vein thrombosis, the number of patients with either of them (VTE) will be larger than the number of individuals who suffer one or the other.

**Figure 2:**
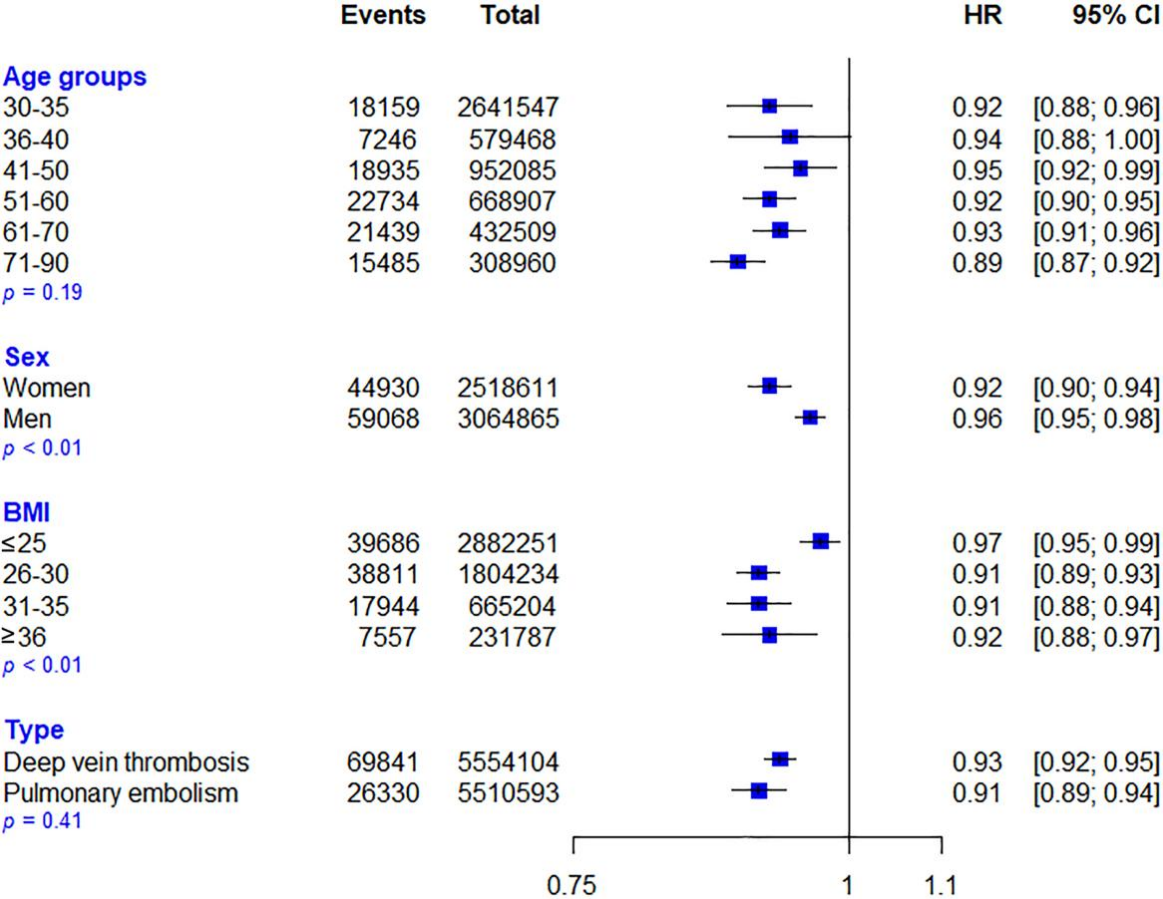
The risk of venous thromboembolism (VTE) per 20 mmHg increase in systolic blood pressure (BP), stratified by age, sex, body mass index (BMI) categories, and type of VTE. Hazard ratio (HR) and 95% confidence interval (CI) are displayed using floating absolute risks and corrected for regression dilution. Models are adjusted for age, sex, BMI, smoking, alcohol, total cholesterol, LDL cholesterol, HDL cholesterol, practice level and anticoagulant prescription during follow-up. *P* = *P*-value for interaction.

Of the 5 588 280 individuals that met the inclusion criteria, 104 017 (1.9%) had a first diagnosis of VTE during the 9.6-year follow-up, of which 26 330 cases were PE, and 69 841 cases were DVT. Participant characteristics stratified by systolic BP categories are shown in *Table 1*. Systolic BP was inversely associated with the risk of VTE in the multi-adjusted model, with each 20 mmHg increase in systolic BP associated with a 7% lower risk of VTE [HR: 0.93, 95% CI (0.92–0.94)] (*Figure 1*). Patients in the highest category of systolic BP (161–180 mmHg) were 18% less likely to be diagnosed with VTE [HR: 0.82, 95% CI: (0.78 to 0.86)]. Subgroup analyses based on age, sex, BMI, and VTE subtype are shown in *Figure 2*. There was no significant heterogeneity across age groups (*P* = 0.19) but relative risks were more substantial for women compared with men (*P* < 0.01), and among participants with BMI > 26 kg/m^2^ than those with lower BMI (*P* < 0.01). Associations were similar for DVT and PE (*P* = 0.41). Sequential adjustments for potential confounders showed that the use of anticoagulants during follow-up did not have any material impact on the association between systolic BP and risk of VTE (see Supplementary material online, *Figure S1*). Sensitivity analyses were associated with no material change in the main results. Detailed results of sensitivity analyses are available in Supplementary material online, *Result S1 and Figure S2*.

**Table 1:** Baseline characteristics of participants in CPRD cohort analysis according to categories of systolic blood pressure

Variables	101–120 mmHg *n* = 1,371,922	121–140 mmHg *n* = 3 680 568	141–160 mmHg *n* = 496 227	161–180 mmHg *n* = 39 563	Total *n* = 5 588 280
**VTE, *n* (%)**	14 501 (1.1)	68 399 (1.9)	19 363 (3.9)	1754 (4.4)	104 017 (1.9)
**Age categories, *n* (%)**					
ȃ30–50	1 276 886 (93)	2 756 101 (75)	139 267 (28)	5012 (13)	4 177 266 (75)
ȃ51–60	64 465 (4.7)	480 637 (13)	116 177 (23)	7967 (20)	669 246 (12)
ȃ61–70	20 598 (1.5)	276 633 (7.5)	123 763 (25)	11 677 (30)	432 671 (7.7)
ȃ71–90	9973 (0.73)	167 197 (4.5)	117 020 (24)	14 907 (38)	309 097 (5.5)
**Age, median (IQR)**	34 (29, 41)	37 (27, 50)	60 (49, 70)	66 (57, 74)	39 (28, 52)
**Women, *n* (%)**	976 538 (71)	1 820 486 (49)	247 246 (50)	23 527 (59)	3 067 797 (55)
**BMI categories, *n* (%)**					
ȃ ≤ 25	755 698 (72)	1 358 863 (50)	108 893 (33)	7808 (32)	2 231 262 (54)
ȃ26–30	223 920 (21)	917 037 (34)	132 424 (40)	9431 (39)	1 282 812 (31)
ȃ31–35	51 249 (4.9)	313 809 (12)	61 437 (18)	4628 (19)	431 123 (10)
ȃ ≥ 35	17 212 (1.6)	134 835 (4.9)	31 053 (9.3)	2492 (10)	185 592 (4.5)
**BMI, median (IQR)**	23 (21, 26)	25 (22, 28)	27 (24, 31)	27 (24, 31)	25 (22, 28)
**Smoking status**					
ȃNon-smoking	693 564 (58)	1 785 905 (57)	221 931 (58)	17 061 (59)	2 718 461 (58)
ȃFormer smoking	119 328 (10)	402 329 (13)	66 598 (17)	5223 (18)	593 478 (13)
ȃCurrent smoking	373 310 (31)	932 527 (30)	96 519 (25)	6767 (23)	1 409 123 (30)
**Plasma lipids**					
ȃTotal	5.0 (4.3, 5.8)	5.4 (4.6, 6.2)	5.6 (4.8, 6.4)	5.6 (4.8, 6.4)	5.4 (4.6, 6.2)
ȃLDL	3.0 (2.4, 3.6)	3.3 (2.6, 3.9)	3.3 (2.6, 4)	3.3 (2.6, 4.0)	3.2 (2.6, 3.9)
ȃHDL	1.4 (1.1, 1.7)	1.3 (1.1, 1.6)	1.3 (1.1, 1.6)	1.4 (1.1, 1.7)	1.3 (1.1, 1.6)
**Follow-up (years), median (IQR)**	9 (4.0, 16)	9.5 (4.6, 17)	11 (6.4, 17)	11 (6.7, 16)	9.6 (4.6, 16)

VTE, venous thromboembolism; BMI, body mass index; LDL, low-density lipoprotein; HDL, high-density lipoprotein; IQR, interquartile range.

### 3.2 Mendelian randomization results

**Figure 3:**
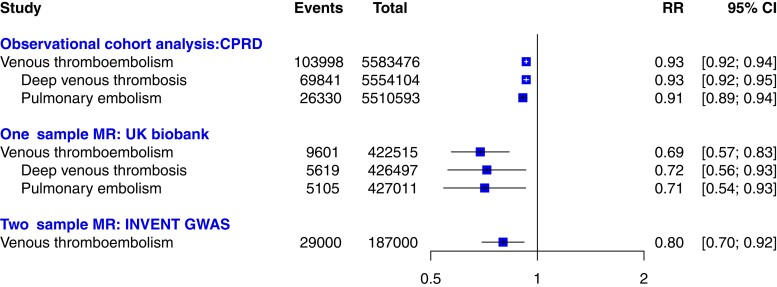
The results of observational cohort and Mendelian randomization studies for the association between systolic blood pressure per 20 mmHg and the risk of venous thromboembolism. Solid squares represent point estimation, and horizontal lines represent 95% confidence intervals. Relative risk (RR), in CPRD cohort analysis indicate hazard ratio and in Mendelian randomization indicate odds ratio. CPRD, Clinical Practice Research Datalink, MR, Mendelian randomization, INVENT, The International Network Against Venous Thrombosis Consortium; GWAS, genome-wide association study.

In the UK Biobank study, we identified 5105 cases of PE and 5619 cases of DVT, or 9601 (2.22%) VTE events (i.e. PE or DVT). The F-statistic from the first-stage regression together with the linear correlation between the polygenic risk score and systolic BP provided evidence that the polygenic risk score was a robust instrumental variable (regression coefficient = 0.199, *P* < 1 × 10^−4^, F-statistic = 5695.1). The distribution of polygenic risk score and measured systolic BP showed in Supplementary material online, *Figure S3*. Using the two-stage least-squares analysis, each 20 mmHg genetically determined higher systolic BP was associated with a 31% lower risk of VTE [odds ratio (OR): 0.69 (95% CI: 0.57–0.83)]. The association was consistent when VTE was stratified into DVT and PE, [OR: 0.72, 95% CI: (0.56–0.93)] and [OR: 0.71, 95% CI: (0.54–0.93)] (*Figure 3*). The positive control results further supported the validity of the analyses, confirming the association between systolic BP and coronary heart disease and stroke (see Supplementary material online, *Figure S4*). The results of sensitivity analysis using an unweighted polygenic risk score led to no change in the main estimations (see Supplementary material online, *Figure S5*). Finally, we found no material change after further adjustments for possible confounders (see Supplementary material online, *Figure S6 and Table S3*). Sensitivity analysis excluding participants with a history of BP treatment and participants with at least one relative to other participants resulted in no material change (see Supplementary material online, *Tables S4 and S5*).

The findings of the one-sample Mendelian randomization were consistent with those of the two-sample analysis (*Figure 3*), which showed strong evidence of a causal association between systolic BP and VTE [OR: 0.80, 95% CI: (0.70–0.92)]. There was little evidence of a non-zero intercept from the MR-Egger regression (intercept *b* = −0.0002, *P* = 0.89), which indicates that genetic pleiotropy did not have a significant effect on the estimation (see Supplementary material online, *Figures S7 and S8*). In addition, the estimates from all Mendelian randomization methods as sensitivity analysis were in line with the main result (see Supplementary material online, *Figure S9*).

## 4. Discussion

In a large-scale population cohort without cardiovascular disease and cancer at baseline, with over 100 000 first episodes of VTE and a median follow-up of about 10 years, we found a 7% lower risk of VTE for each 20 mmHg higher systolic BP. The association was comparable when we examined PE and DVT separately and persisted after taking into account age and other factors, including anticoagulant treatment during follow-up. Furthermore, when we further investigated the observed association by conducting Mendelian randomization analysis in separate data sets with genetic information, we found an observable association between genetically determined elevated systolic BP and risk of VTE. Taken together, our findings suggest that the association between higher systolic BP and lower risk of VTE and its subtypes is likely to be causal.

Considering that the magnitude of relative risks observed in our cohort study was small, we were concerned about the possibility of uncontrolled confounding. Indeed, we were surprised about the absence of any interaction by age, given the consistency of evidence from observational studies that have investigated associations between BP and other types of cardiovascular disease. The typically observed attenuation of HRs with increasing age in those studies^19,40^ is usually explained by the accumulation of several risk factors among the elderly and diminishing relative contribution of each of them. The lack of heterogeneity by age in our cohort analysis raised the possibility that the overall negative but weak associations might be spurious and due to residual confounding or reverse causality. To investigate this further, we conducted two Mendelian randomization studies, which are not prone to reverse causation and confounding,^41^ and found strong evidence for the effect of systolic BP on VTE.

The findings of our studies are in keeping with one IPD meta-analysis^15^ of prospective cohort studies that showed that increased BP was associated with a lower risk of VTE. This association was unexpected as a previous meta-analysis^14^ had shown an association in the opposite direction. This made the authors of the IPD meta-analysis hypothesize that the inverse correlation between BP and VTE risk might have been due to differential use of anticoagulant treatment during follow-up. In our study, we were able to assess this hypothesis more directly. We found that adjustment for anticoagulant therapy had no significant impact on the strength or direction of the association between systolic BP and VTE. Besides, our findings of a relatively small effect size might also explain why in two recent cohort studies, there was no clear evidence of association.^16^

Considering the strong evidence in our study in favour of the causal nature of the observed association, it is worth considering the possible mechanisms for such an effect. Virchow’s triad describes the cause of VTE based on three underlying factors, including vessel wall damage, hypercoagulability, and circulatory stasis, specifically low BP.^42^ Low BP could, therefore, lead to reduced flow of oxygenated blood in veins, predisposing the endothelium to hypoxaemia. A general property of endothelial cells is that they get activated by hypoxia, metabolic stress, and inflammatory cytokines. Hypoxaemic endothelium could then lead to inflammation and expression of adhesion molecules.^43–45^ This could then trigger the coagulation cascade via the extrinsic pathway.^42,46^ Further experimental study on the biological mechanisms of the association is warranted.

This study has several strengths in comparison to previous reports. Firstly, our cohort had a large sample size and included a large number of VTE cases, which then increased the power to detect association across the whole spectrum of systolic BP and to perform subgroup analysis. Secondly, time-varying adjustment for anticoagulant treatment during follow-up and other confounders, as well as adjustment for cohort effect and regression dilution bias, allowed addressing some of the limitations of previous studies. Finally, the main strength was the addition of Mendelian randomization analyses based on genetic instruments that served as a proxy for elevated systolic BP, thus mitigating the risk of residual confounding and reverse causality.

On the other hand, this study has some limitations worth mentioning. Our cohort study was based on routinely collected data from linked electronic health records, which may be prone to measurement errors. With regards to our exposure variable, this issue was addressed by using the repeated measurement of systolic BP before the VTE event, and their correction for regression dilution bias. The outcome was defined using an externally validated algorithm.^20^ However, we acknowledge a degree of misclassification because outcome definition relied mainly on data retrieved from linked electronic health records with no data available for sub-classification of cases as provoked or unprovoked VTE. However, the previous IPD meta-analysis that also reported a negative association between systolic BP and VTE found no evidence of interaction between provoked and unprovoked cases of VTE. Finally, our Mendelian randomization analysis assumed that the genetic variants selected as a proxy for elevated systolic BP influence the outcome (i.e. VTE) only through systolic BP (i.e. exposure of interest). Although we cannot be sure that the genetic variants included in polygenic risk score do not have pleiotropic effects, we did not find any evidence in favour of pleiotropy.

## 5. Conclusion

We found an increased risk of VTE with lower BP. This association was independently confirmed in two Mendelian randomization analyses supporting a possible causal link. The benefits of BP reduction are likely to outweigh the harms in most patient groups, but in people with predisposing factors for VTE, further BP reduction should be made cautiously.

## Supplementary material

Supplementary material is available at *Cardiovascular Research* online.

## Supplementary Material

cvac135_Supplementary_Data

## Data Availability

Data can be obtained directly from CPRD subject to the custodian’s policies for scientific, data governance, and financial approvals (see www.cprd.com). All bona fide researchers can apply to use the UK Biobank data set for health-related research. A guide to access is also provided on the UK Biobank website (see www.ukbiobank.ac.uk).

## Notes

Translational perspective In a large-scale population cohort, with over 100 000 first episodes of venous thromboembolism (VTE) and a median follow-up of about 10 years, we found a 7% higher risk of VTE for each 20 mmHg lower systolic blood pressure (BP). The association was comparable when we examined pulmonary embolism and deep venous thrombosis separately, and persisted after taking into account age and other factors, including anticoagulant treatment during follow-up. These results were confirmed using two independent Mendelian randomization studies. Although the beneficial effects of BP-lowering are likely to outweigh any harms in most patient groups, clinicians should be aware of the potential risk of VTE from anti-hypertensive therapy, in particular in people who have predisposing factors for VTE.
