# Supplementary material for: Blood pressure and risk of venous thromboembolism: a cohort analysis of 5.5 million UK adults and Mendelian randomization studies
Source: Cardiovasc Res. 2022 Aug 29;119(3):835–42. doi: 10.1093/cvr/cvac135 (PMC10153414; doi:10.1093/cvr/cvac135)
Supplement: cvac135_Supplementary_Data [file cvac135_supplementary_data.docx]

**Supplementary materials**

**Blood pressure and risk of venous thromboembolism: a cohort analysis of 5.5 million UK adults and Mendelian randomization studies**

Milad Nazarzadeh, Zeinab Bidel, Hamid Mohseni, Dexter Canoy, Ana-Catarina Pinho-Gomes, Abdelaali Hassaine, Abbas Dehghan, David-Alexandre Tregouet, Nicholas L. Smith, Kazem Rahimi, INVENT Consortium

Contents

[Methods S1. Detailed methods of CPRD cohort analysis. 3](#_Toc102129871)

[Method S2. Details of constructing polygenic risk scores from GWAS studies. 5](#_Toc102129872)

[Results S1. Detailed results of CPRD observational cohort analysis. 6](#_Toc102129873)

[Table S1. Diagnostic codes for the definition of venous thromboembolism and its subtypes. 7](#_Toc102129874)

[Table S2. The characteristics of selected genetic variants for systolic blood pressure. 8](#_Toc102129875)

[Table S3. Sensitivity analysis adjusting the one-sample Mendelian randomization for comorbid cardiometabolic conditions. 18](#_Toc102129876)

[Table S4. Sensitivity analysis excluding participants with a history of blood pressure treatment use in one-sample Mendelian randomization. 18](#_Toc102129877)

[Table S5. Sensitivity analysis excluding participants with at least one relative to other participants in one-sample Mendelian randomization. 18](#_Toc102129878)

[Figure S1. The hazard ratio for venous thromboembolism per 20 mmHg increase in systolic blood pressure with progressive adjustment. 19](#_Toc102129879)

[Figure S2. Sensitivity analysis of the association between systolic blood pressure and the risk of venous thromboembolism stratified based on objective criteria for outcome definition. 19](#_Toc102129880)

[Figure S3. The distribution of genetic risk score and measured systolic blood pressure in the one-sample Mendelian randomization. 20](#_Toc102129881)

[Figure S4. Positive control analysis to test the validity of the instrumental variable. 21](#_Toc102129882)

[Figure S5. Sensitivity analysis using unweighted polygenic risk score to check the robustness of the weighting approach 21](#_Toc102129883)

[Figure S6. Sensitivity analysis to assess the effect of adjusting for potential confounders on the main estimations. 22](#_Toc102129884)

[Figure S7. Scatter plot of 276 variants associated with systolic blood pressure and their effect on venous thromboembolism. 22](#_Toc102129885)

[Figure S8. Funnel plot of 276 variants, showing each variant causal estimate against instrument strength. 23](#_Toc102129886)

[Figure S9. Sensitivity analysis methods of two-sample Mendelian randomization to assess the causal association between systolic blood pressure and risk of venous thromboembolism. 24](#_Toc102129887)

[References 25](#_Toc102129888)

## Methods S1. Detailed methods of CPRD cohort analysis.

The Clinical Practice Research Datalink (CPRD) database contains anonymized patient data from 674 general practices in the United Kingdom (UK), covering approximately 7% of the current UK population, and it is broadly representative of the UK population by age, sex and ethnicity.^1^ It links primary care records with discharge diagnosis from Hospital Episode Statistics (HES), and mortality data from national death registries (Office for National Statistics) using a coding system based on the 10^th^ version of the International Classification of Diseases (ICD).^2^ It is a very comprehensive prospective primary care database whose validity has been reviewed elsewhere.^3,4^ The scientific approval for this study was given by the CPRD Independent Scientific Advisory Committee (ISAC).

The data used in this study were shown as numbers and percentages by cross-tabulation. The Cox proportional hazard model with 9.6 years of follow-up was used to estimate the multivariable-adjusted hazard ratio (HR) for venous thromboembolism (VTE) by systolic blood pressure stratum. Floating absolute risks, which do not require the selection of an arbitrary baseline group for display of confidence intervals (CI), were used to present HRs for VTE for each systolic blood pressure category.^5^ In floating absolute risks, rather than using a single risk factor level as a reference category, each level is given a 'floated' variance that expresses risk uncertainty without referencing another level. This approach does not change the estimated relative risks, but it does generate standard errors and confidence intervals that allow meaningful comparisons between any two groups, even if neither is the baseline group.^6^ Due to instrument error, intra-subject variation, or both, random measurement error generally occurs when systolic blood pressure measurements fluctuate randomly within their true values.^7^ To account for the measurement error of single blood pressure measurement and other time-dependent fluctuations of systolic blood pressure, we used multiple systolic blood pressure measurements to calculate ‘usual’ values that were corrected for regression dilution. We used generalized estimating equations to regress serial systolic blood pressure measurements within the median follow-up (mean of 6.7 measurements per patient) on the baseline systolic blood pressure. The estimated regression dilution ratio for SBP was 0.56.

We considered systolic blood pressure both as a categorical and continuous variable in the Cox model. In the continuous model, SBP was analyzed in 20 mmHg increments (approximately one standard deviation increase). In the categorical model, SBP categories were defined as 101 to 120, 121 to 140, 141 to 160, and 161 to 180 mmHg.

The model was adjusted for the following baseline variables: sex, age, body mass index (BMI, kg/m^2^), smoking status (current, never and former smoking), lipid profile (total, LDL, HDL cholesterol), anticoagulant treatment as a time-varying variable during follow-up. Anticoagulant drugs are obtained using the British National Formulary (BNF) codes in the therapy table. We also conducted the stratified analysis for the practice-level index of multiple deprivation (IMD) quintiles. The closest measurement within two years of the baseline blood pressure measurements was used as the baseline value for covariates. The covariate was considered as missing if no measurement was available within this time frame.

Several sensitivity analyses were conducted on the data. First, the first two and three years of study follow-up were excluded, and the analysis was repeated so that potential reverse causation could be prevented. Second, the models were adjusted for year of the initial systolic blood pressure measurement, as a categorical variable (1985 to 1989, 1990 to 1994, 1995 to 1999, 2000 to 2004, 2005 to 2009, and 2010 to 2013) in order to address potential cohort effects. Third, total cholesterol, LDL, and HDL as covariates were excluded from the primary analyses due to their high proportion of missing values, and then the impact of over-adjustment was assessed. Finally, subgroup analyses were conducted based on age categories, sex, BMI categories, subtype of VTE (deep venous thrombosis [DVT] and pulmonary embolism [PE]) and different diagnostic criteria for VTE (imaging test, anticoagulant prescription or death and source of patients).

## Method S2. Details of constructing polygenic risk scores from GWAS studies.

Because of overlap between the selected GWAS and UK biobank, ^8^ and to avoid bias due to sample overlap, ^9^ we extracted the corresponding beta coefficients and standard errors from the International Consortium for Blood Pressure GWAS (ICBP), which did not include the UK biobank^10^ and therefore provided a non-overlapping sample. ICBP is a GWAS meta-analysis including about 200,000 European population, and its estimations were adjusted for sex, age, age-squared, BMI, within-cohort stratification, and also for blood pressure-lowering medications use.^10^ To construct polygenic risk scores for systolic blood pressure, we used the following approach. First, each variant was recoded additively (0, 1, and 2) according to the number of alleles that increase the systolic blood pressure. Then, each variant was weighed according to the regression coefficient obtained from the GWAS meta-analysis to give more weight to variants with stronger effects. Finally, a weighted polygenic risk score was constructed using the following formula:

(beta_1_ × varinat_1_) + (beta_2_ × varinat_2_) + … + (beta_n_ × varinat_n_)

Where “beta _i_” was the regression coefficient associated with “variant _i_” and obtained from the ICBP GWAS study. ^10^

## Results S1. Detailed results of CPRD observational cohort analysis.

A total of 6,613,644 individuals aged between 30 and 90 years with at least one blood pressure measurement were considered initially for the analysis. After excluding individuals at baseline with previous diagnosis of cardiovascular disease (n = 380,785), cancer (n = 129,087) or VTE (n = 53,651), taking lipid-lowering or anti-hypertensive medications (n =184,546), or with less than one year follow-up (n = 265,225), a total of 5,588,280 individuals were included for analysis. Median follow-up was 9.6 (range 4.6 to 16) years. Overall, participants with higher SBP were more likely to be older and female and to have concurrently obesity, smoking, and elevated cholesterol. Furthermore, VTE was more common in people with higher SBP.

*Sensitivity analysis results*

Sensitivity analyses produced very similar results to the main analyses. After excluding all the VTE events during the first two years of follow-up, the HR for each 20 mmHg increase in SBP was 0.94 (95% CI [0.93 to 0.96]). Next, excluding patients with less than three years of follow-up yielded HR equal to 0.95 [95% CI [0.93 to 0.96]). Exclusion of the total cholesterol, LDL, and HDL from the model had little effect on the association (HR 0.93 (95% CI [0.92 to 0.94]). The potential impact of over-adjustment was assessed by a progressive adjustment for covariates, in which consecutively adjustment had little effect on the estimated HRs between SBP and risk of VTE (**Figure S1**). Besides, removing the adjustment for the year of SBP measurement did not change HR substantially (0.91, 95% CI [0.90 to 0.92]), and adjusting for atrial fibrillation during follow-up also did not influence the HR (0.93, 95% CI [0.92 to 0.94]). Finally, stratified analysis based on having imaging test for diagnosis of VTE showed that the association for SBP was stronger in participants who had VTE diagnosed with an objective imaging test than in participants without imaging confirmation, but the interaction was not significant (p = 0.10) (**Figure S2**). Also, further stratification based on validation of diagnostic criteria using anticoagulant use or death within 30 days after VTE event and source of patient diagnosis (out-patient versus in-patient) did not show any material difference between strata (**Figure S2**).

| Table S1. Diagnostic codes for the definition of venous thromboembolism and its subtypes. | | |
| --- | --- | --- |
|  | **ICD10** | Subcategory |
| I80.1 | Phlebitis and thrombophlebitis of femoral vein | DVT |
| I80.2 | Phlebitis and thrombophlebitis of other deep vessels of lower extremities | DVT |
| I80.3 | Phlebitis and thrombophlebitis of lower extremities, unspecified | DVT |
| I80.0 | Phlebitis and thrombophlebitis of superficial vessels of lower extremities | DVT |
| I80.8 | Phlebitis and thrombophlebitis of other sites | DVT |
| I80.9 | Phlebitis and thrombophlebitis of unspecified sites | DVT |
| I81 | Portal vein thrombosis | DVT |
| I82.0 | Budd-Chiari syndrome | DVT |
| I82.2 | Embolism and thrombosis of vena cava | DVT |
| I82.3 | Embolism and thrombosis of renal vein | DVT |
| I82.4 | Acute embolism and thrombosis of deep veins of lower extremity | DVT |
| I82.6 | Acute embolism and thrombosis of veins of upper extremity | DVT |
| I82.8 | Embolism and thrombosis of other specified veins | DVT |
| I82.9 | Embolism and thrombosis of unspecified vein | DVT |
| I67.6 | Nonpyogenic thrombosis of intracranial venous system | DVT |
| O22.5 | Cerebral venous thrombosis in pregnancy | DVT |
| O22.3 | Deep phlebothrombosis in pregnancy | DVT |
| O87.1 | Deep phlebothrombosis in the puerperium | DVT |
| I26.0 | Pulmonary embolism with mention of acute cor pulmonale | PE |
| I26.9 | Pulmonary embolism without mention of acute cor pulmonale | PE |
| I26.02 | Saddle embolus of pulmonary artery with acute cor pulmonale | PE |
| I26.09 | Other pulmonary embolism with acute cor pulmonale | PE |
| I26.92 | Saddle embolus of pulmonary artery without acute cor pulmonale | PE |
| I26.99 | Other pulmonary embolism without acute cor pulmonale | PE |
| **ICD 9** | | |
| 4510 | Phlebitis and thrombophlebitis of superficial vessels of lower extremity | DVT |
| 4511 | Phlebitis and thrombophlebitis of deep vessels of lower extremity | DVT |
| 4512 | Phlebitis and thrombophlebitis of lower extremities, unspecified | DVT |
| 4518 | Phlebitis and thrombophlebitis of other specified sites | DVT |
| 4519 | Phlebitis and thrombophlebitis of unspecified site | DVT |
| 4532 | Embolism and thrombosis of vena cava | DVT |
| 4538 | Embolism and thrombosis of other specified veins | DVT |
| 6712 | Superficial thrombophlebitis in pregnancy and the puerperium | DVT |
| 6714 | Deep phlebothrombosis, postpartum | DVT |
| 4151 | Pulmonary embolism | PE |
|  | **Self-report (UK Biobank only)** |  |
| 1094 | Deep venous thrombosis | DVT |
| 1093 | Pulmonary embolism | PE |
| ICD: The International Classification of Diseases, DVT: Deep vein thrombosis, PE: pulmonary embolism | | |

| Table S2. The characteristics of selected genetic variants for systolic blood pressure. | | | | | | | | | |  |
| --- | --- | --- | --- | --- | --- | --- | --- | --- | --- | --- |
| SNP * | Chromosome | Position (GRCh37) | Allele1 | Allele2 | Freq1† | Effect † | Standard error † | P-value† | Source * | Genome-wide association with cardiovascular disease or risk factors  (based on the The NHGRI-EBI database; P-value <5 × 10−8) |
| rs3737801 | 1 | 27960832 | c | g | 0.9142 | 0.4246 | 0.0954 | 8.67E-06 | Novel:one-stage design | - |
| rs11210029 | 1 | 41865293 | a | g | 0.625 | -0.1608 | 0.0476 | 0.000728 | Novel:one-stage design | - |
| rs11579440 | 1 | 49052423 | t | c | 0.8468 | 0.2794 | 0.0653 | 1.86E-05 | Novel:one-stage design | - |
| rs10923038 | 1 | 88651771 | a | c | 0.6166 | 0.1279 | 0.0481 | 0.00781 | Novel:one-stage design | - |
| rs76719272 | 1 | 156129796 | t | c | 0.1309 | -0.2747 | 0.0727 | 0.00016 | Novel:two-stage design | - |
| rs1043069 | 1 | 180859368 | t | g | 0.6225 | 0.2696 | 0.0478 | 1.72E-08 | Novel:two-stage design | - |
| rs4651224 | 1 | 184585182 | t | c | 0.4518 | 0.144 | 0.047 | 0.002185 | Novel:two-stage design | - |
| rs12042924 | 1 | 197297417 | t | c | 0.5202 | -0.1372 | 0.0465 | 0.003202 | Novel:two-stage design | - |
| rs33996239 | 1 | 203109801 | t | c | 0.0577 | -0.427 | 0.1058 | 5.43E-05 | Novel:two-stage design | - |
| rs7555285 | 1 | 209970355 | c | g | 0.7951 | 0.173 | 0.0565 | 0.002212 | Novel:two-stage design | - |
| rs260508 | 1 | 2187085 | t | g | 0.6167 | 0.1696 | 0.0477 | 0.000377 | Novel:two-stage design | - |
| rs2807337 | 1 | 22577371 | t | c | 0.3721 | 0.1938 | 0.0478 | 5.02E-05 | Novel:two-stage design | - |
| rs4926499 | 1 | 249155909 | c | g | 0.8262 | 0.2922 | 0.0752 | 0.000102 | Novel:two-stage design | - |
| rs79598313 | 1 | 27284913 | t | c | 0.0275 | 0.5126 | 0.1518 | 0.00073 | Novel:two-stage design | CRP, diabetes, LDL, |
| rs839755 | 1 | 43856410 | a | c | 0.6224 | -0.1877 | 0.047 | 6.55E-05 | Novel:two-stage design | - |
| rs7514579 | 1 | 94051350 | a | c | 0.7765 | 0.3027 | 0.0559 | 6.16E-08 | Novel:two-stage design | - |
| rs17396055 | 1 | 94730954 | a | g | 0.3317 | -0.1525 | 0.049 | 0.001874 | Novel:two-stage design | - |
| rs880315 | 1 | 10796866 | t | c | 0.652 | -0.5218 | 0.0499 | 1.33E-25 | Previously reported | TG, LDL, Stroke, AF, Smoking, Albumin, |
| rs4846049 | 1 | 11850365 | t | g | 0.3264 | -0.4146 | 0.0489 | 2.44E-17 | Previously reported | - |
| rs17367504 | 1 | 11862778 | a | g | 0.8444 | 0.7774 | 0.0639 | 4.81E-34 | Previously reported | Mean platelet volume, smoking |
| rs5068 | 1 | 11905974 | a | g | 0.9387 | 1.0914 | 0.0989 | 2.53E-28 | Previously reported | Smoking |
| rs3820068 | 1 | 15798197 | a | g | 0.7977 | 0.3361 | 0.0596 | 1.69E-08 | Previously reported | - |
| rs7515635 | 1 | 42408070 | t | c | 0.4684 | 0.2382 | 0.0463 | 2.70E-07 | Previously reported |  |
| rs10922502 | 1 | 89360158 | a | g | 0.6407 | -0.2283 | 0.0483 | 2.32E-06 | Previously reported |  |
| rs55732192 | 2 | 162278233 | t | g | 0.0962 | -0.2807 | 0.0798 | 0.000433 | Novel:one-stage design |  |
| rs6712203 | 2 | 165557318 | t | c | 0.3779 | -0.1943 | 0.0477 | 4.57E-05 | Novel:one-stage design | Alanine aminotransferase levels |
| rs11694601 | 2 | 174949358 | a | g | 0.5927 | -0.1422 | 0.047 | 0.00249 | Novel:one-stage design |  |
| rs1837164 | 2 | 178716601 | a | t | 0.3753 | 0.186 | 0.0472 | 8.27E-05 | Novel:one-stage design |  |
| rs296797 | 2 | 201102905 | t | c | 0.4142 | 0.2067 | 0.0467 | 9.68E-06 | Novel:one-stage design |  |
| rs1047891 | 2 | 211540507 | a | c | 0.3243 | -0.1647 | 0.0511 | 0.00127 | Novel:one-stage design | CKD, Platelet count, WBC counts, RBC counts, Fat-free mass, etc. |
| rs10189186 | 2 | 53025757 | a | g | 0.5357 | 0.1752 | 0.0459 | 0.000135 | Novel:one-stage design |  |
| rs28377357 | 2 | 112769721 | a | g | 0.3 | -0.1588 | 0.0502 | 0.001572 | Novel:two-stage design |  |
| rs6723509 | 2 | 122000745 | t | c | 0.8617 | 0.2553 | 0.0677 | 0.000162 | Novel:two-stage design |  |
| rs72844590 | 2 | 138421227 | t | g | 0.1441 | 0.0856 | 0.0692 | 0.2158 | Novel:two-stage design |  |
| rs79523138 | 2 | 161368213 | a | g | 0.8849 | -0.3083 | 0.0749 | 3.83E-05 | Novel:two-stage design |  |
| rs6739913 | 2 | 185033065 | a | g | 0.7095 | -0.1523 | 0.0506 | 0.002638 | Novel:two-stage design |  |
| rs28558491 | 2 | 187816321 | t | c | 0.7362 | -0.1935 | 0.0531 | 0.000265 | Novel:two-stage design |  |
| rs67720684 | 2 | 18975439 | a | c | 0.2295 | 0.0834 | 0.0546 | 0.1269 | Novel:two-stage design |  |
| rs12694277 | 2 | 213188795 | t | c | 0.2914 | -0.219 | 0.051 | 1.77E-05 | Novel:two-stage design |  |
| rs1044822 | 2 | 230629138 | t | c | 0.142 | -0.2655 | 0.0657 | 5.38E-05 | Novel:two-stage design |  |
| rs139354822 | 2 | 242344695 | t | c | 0.9675 | 0.4794 | 0.1554 | 0.002042 | Novel:two-stage design |  |
| rs35590893 | 2 | 43716933 | a | g | 0.2716 | -0.1215 | 0.0515 | 0.01822 | Novel:two-stage design |  |
| rs6545155 | 2 | 50429861 | t | c | 0.7852 | 0.2182 | 0.0559 | 9.56E-05 | Novel:two-stage design |  |
| rs2920899 | 2 | 55279681 | t | g | 0.7851 | 0.1653 | 0.0573 | 0.003886 | Novel:two-stage design |  |
| rs72816333 | 2 | 60096560 | a | t | 0.8277 | 0.254 | 0.0606 | 2.79E-05 | Novel:two-stage design |  |
| rs2300481 | 2 | 66782467 | t | c | 0.3886 | 0.2043 | 0.0472 | 1.50E-05 | Novel:two-stage design | BMI |
| rs1446468 | 2 | 164963486 | t | c | 0.4512 | -0.487 | 0.0468 | 2.26E-25 | Previously reported | Smoking |
| rs6712094 | 2 | 165043460 | a | g | 0.7296 | 0.42 | 0.0525 | 1.17E-15 | Previously reported |  |
| rs6749447 | 2 | 169041386 | t | g | 0.7323 | -0.067 | 0.0523 | 0.2008 | Previously reported |  |
| rs6434404 | 2 | 191494411 | a | g | 0.3247 | 0.2228 | 0.0498 | 7.53E-06 | Previously reported |  |
| rs1344653 | 2 | 19730845 | a | g | 0.4961 | -0.1568 | 0.0456 | 0.00058 | Previously reported |  |
| rs55780018 | 2 | 208526140 | t | c | 0.548 | -0.3278 | 0.0488 | 1.85E-11 | Previously reported |  |
| rs2972146 | 2 | 227100698 | t | g | 0.6362 | 0.2486 | 0.0476 | 1.76E-07 | Previously reported | TG, CAD, HDL |
| rs55701159 | 2 | 25139596 | t | g | 0.887 | 0.2999 | 0.0742 | 5.27E-05 | Previously reported |  |
| rs1275988 | 2 | 26914364 | t | c | 0.6055 | -0.5157 | 0.0466 | 1.83E-28 | Previously reported | CVD, smoking |
| rs9678851 | 2 | 27887034 | a | c | 0.559 | -0.1135 | 0.0474 | 0.01662 | Previously reported |  |
| rs7562 | 2 | 28635740 | t | c | 0.5297 | 0.1555 | 0.047 | 0.00093 | Previously reported |  |
| rs13420463 | 2 | 37517566 | a | g | 0.7775 | 0.2751 | 0.0555 | 7.19E-07 | Previously reported |  |
| rs262986 | 3 | 183435713 | a | g | 0.4712 | -0.2288 | 0.0468 | 1.01E-06 | Novel:one-stage design |  |
| rs1882289 | 3 | 114461208 | a | g | 0.8814 | -0.2919 | 0.0708 | 3.78E-05 | Novel:two-stage design |  |
| rs9875380 | 3 | 132780356 | t | c | 0.4619 | -0.2472 | 0.0457 | 6.21E-08 | Novel:two-stage design |  |
| rs863930 | 3 | 135949737 | t | g | 0.4671 | -0.191 | 0.046 | 3.28E-05 | Novel:two-stage design |  |
| rs78151625 | 3 | 158316726 | t | c | 0.831 | -0.222 | 0.0618 | 0.000329 | Novel:two-stage design |  |
| rs189267552 | 3 | 20073193 | a | t | 0.0141 | -0.7415 | 0.2166 | 0.000618 | Novel:two-stage design | Serum creatinine levels |
| rs12638085 | 3 | 30405936 | a | t | 0.3525 | 0.2514 | 0.0486 | 2.30E-07 | Novel:two-stage design |  |
| rs6788984 | 3 | 41107173 | a | g | 0.858 | 0.3015 | 0.066 | 4.92E-06 | Novel:two-stage design |  |
| rs6774721 | 3 | 49381898 | a | g | 0.1465 | -0.2171 | 0.0689 | 0.001627 | Novel:two-stage design |  |
| rs9857362 | 3 | 74710462 | a | c | 0.5249 | 0.1736 | 0.0473 | 0.000241 | Novel:two-stage design |  |
| rs347591 | 3 | 11290122 | t | g | 0.6625 | 0.2842 | 0.0489 | 6.31E-09 | Previously reported |  |
| rs11128722 | 3 | 14958126 | a | g | 0.5628 | -0.2518 | 0.047 | 8.53E-08 | Previously reported |  |
| rs143112823 | 3 | 154707967 | a | g | 0.076 | -0.4019 | 0.0949 | 2.29E-05 | Previously reported |  |
| rs3097937 | 4 | 124794644 | a | t | 0.8075 | 0.2388 | 0.0587 | 4.77E-05 | Novel:one-stage design |  |
| rs6823767 | 4 | 151295085 | t | c | 0.7227 | -0.1566 | 0.0528 | 0.003027 | Novel:one-stage design |  |
| rs7439567 | 4 | 138464842 | t | c | 0.4157 | 0.245 | 0.0474 | 2.39E-07 | Novel:two-stage design |  |
| rs17035181 | 4 | 157678511 | t | g | 0.8549 | 0.2169 | 0.0653 | 0.000898 | Novel:two-stage design |  |
| rs2610990 | 4 | 18008232 | a | g | 0.2693 | -0.2325 | 0.0523 | 8.86E-06 | Novel:two-stage design | BMI |
| rs231708 | 4 | 2694773 | c | g | 0.6983 | -0.2643 | 0.0499 | 1.19E-07 | Novel:two-stage design |  |
| rs12511987 | 4 | 46595623 | t | g | 0.8224 | -0.2456 | 0.0614 | 6.26E-05 | Novel:two-stage design |  |
| rs1347345 | 4 | 95938386 | a | g | 0.6206 | -0.1645 | 0.0478 | 0.000583 | Novel:two-stage design |  |
| rs13112725 | 4 | 106911742 | c | g | 0.7682 | 0.397 | 0.0557 | 1.01E-12 | Previously reported |  |
| rs2291435 | 4 | 38387395 | t | c | 0.5248 | -0.2419 | 0.0463 | 1.74E-07 | Previously reported |  |
| rs2014912 | 4 | 86715670 | t | c | 0.1515 | 0.5122 | 0.0644 | 1.80E-15 | Previously reported |  |
| rs1650911 | 5 | 141740620 | c | g | 0.7619 | 0.2465 | 0.0584 | 2.42E-05 | Novel:one-stage design |  |
| rs12153395 | 5 | 179411477 | a | g | 0.1133 | -0.2602 | 0.0764 | 0.000661 | Novel:one-stage design |  |
| rs4957026 | 5 | 361148 | a | g | 0.3503 | 0.2214 | 0.0497 | 8.29E-06 | Novel:one-stage design |  |
| rs6875372 | 5 | 64079015 | a | t | 0.5154 | 0.2228 | 0.0459 | 1.18E-06 | Novel:one-stage design |  |
| rs1871190 | 5 | 97953719 | t | g | 0.3472 | 0.1658 | 0.0495 | 0.000805 | Novel:one-stage design |  |
| rs62373688 | 5 | 127352807 | a | t | 0.1259 | 0.3593 | 0.0714 | 4.76E-07 | Novel:two-stage design |  |
| rs10069690 | 5 | 1279790 | t | c | 0.2583 | 0.3827 | 0.0627 | 1.03E-09 | Novel:two-stage design | Albumin levels, Cancer |
| rs702395 | 5 | 140086677 | t | c | 0.4369 | 0.2367 | 0.0468 | 4.31E-07 | Novel:two-stage design |  |
| rs74774746 | 5 | 33411769 | c | g | 0.2639 | -0.1177 | 0.0541 | 0.02953 | Novel:two-stage design |  |
| rs13179413 | 5 | 55868097 | t | c | 0.2775 | 0.1383 | 0.0544 | 0.01098 | Novel:two-stage design | CVD |
| rs3121685 | 5 | 65662133 | t | c | 0.4815 | -0.2015 | 0.046 | 1.17E-05 | Novel:two-stage design |  |
| rs246973 | 5 | 68007803 | t | c | 0.2833 | 0.1984 | 0.0509 | 9.60E-05 | Novel:two-stage design |  |
| rs709668 | 5 | 96174186 | a | g | 0.1965 | -0.2755 | 0.0576 | 1.72E-06 | Novel:two-stage design |  |
| rs10077885 | 5 | 114390121 | a | c | 0.498 | -0.2465 | 0.0484 | 3.54E-07 | Previously reported | Smoking |
| rs1008058 | 5 | 122435627 | a | g | 0.1183 | 0.3142 | 0.0766 | 4.12E-05 | Previously reported |  |
| rs13359291 | 5 | 122476457 | a | g | 0.1654 | 0.4005 | 0.062 | 1.06E-10 | Previously reported |  |
| rs6595838 | 5 | 127868199 | a | g | 0.2891 | 0.2361 | 0.0507 | 3.14E-06 | Previously reported |  |
| rs11953630 | 5 | 157845402 | t | c | 0.3694 | -0.4463 | 0.0501 | 5.15E-19 | Previously reported | smoking |
| rs1421811 | 5 | 32714270 | c | g | 0.6116 | 0.4743 | 0.0477 | 2.46E-23 | Previously reported | BMI |
| rs1173771 | 5 | 32815028 | a | g | 0.3976 | -0.5227 | 0.0468 | 6.04E-29 | Previously reported | BMI, height |
| rs10059921 | 5 | 87514515 | t | g | 0.0846 | -0.3732 | 0.0919 | 4.89E-05 | Previously reported |  |
| rs7765526 | 6 | 147713764 | a | g | 0.4682 | 0.2317 | 0.047 | 8.11E-07 | Novel:one-stage design | WBC counts |
| rs9449350 | 6 | 82281417 | t | c | 0.673 | -0.2333 | 0.0488 | 1.72E-06 | Novel:one-stage design |  |
| rs9401090 | 6 | 119113317 | t | c | 0.7538 | 0.2512 | 0.054 | 3.32E-06 | Novel:two-stage design |  |
| rs10782230 | 6 | 126228512 | a | g | 0.4907 | 0.2787 | 0.0459 | 1.27E-09 | Novel:two-stage design | Urate levels, RBC count |
| rs9885632 | 6 | 131311909 | t | c | 0.7338 | 0.245 | 0.052 | 2.42E-06 | Novel:two-stage design |  |
| rs7763294 | 6 | 140383733 | t | g | 0.3169 | -0.2059 | 0.0493 | 2.95E-05 | Novel:two-stage design |  |
| rs2745599 | 6 | 1613686 | a | g | 0.5476 | 0.2128 | 0.0513 | 3.30E-05 | Novel:two-stage design | Waist-hip ratio |
| rs9368222 | 6 | 20686996 | a | c | 0.2767 | 0.1639 | 0.0511 | 0.001338 | Novel:two-stage design | Diabetes, CVD |
| rs6911827 | 6 | 22130601 | t | c | 0.4623 | 0.152 | 0.0473 | 0.001295 | Previously reported | Hematocrit, Hemoglobin |
| rs2270860 | 6 | 43270151 | t | c | 0.3092 | 0.2966 | 0.05 | 3.09E-09 | Previously reported | Smoking |
| rs10948071 | 6 | 43280713 | t | c | 0.5993 | -0.2074 | 0.0465 | 8.13E-06 | Previously reported | Smoking |
| rs1563788 | 6 | 43308363 | t | c | 0.2937 | 0.3062 | 0.0501 | 9.79E-10 | Previously reported | BMI |
| rs78648104 | 6 | 50683009 | t | c | 0.8985 | -0.3571 | 0.083 | 1.69E-05 | Previously reported | Cystatin C levels, BMI |
| rs35410524 | 6 | 96885405 | t | c | 0.1917 | 0.2999 | 0.0588 | 3.38E-07 | Previously reported |  |
| rs1870735 | 7 | 155744303 | c | g | 0.4548 | 0.2137 | 0.0486 | 1.08E-05 | Novel:one-stage design |  |
| rs12979 | 7 | 24738164 | c | g | 0.8745 | 0.2241 | 0.0693 | 0.001227 | Novel:one-stage design |  |
| rs34072724 | 7 | 130432469 | a | g | 0.4828 | -0.1967 | 0.0465 | 2.37E-05 | Novel:two-stage design | VLDL |
| rs12703989 | 7 | 140238048 | a | g | 0.494 | 0.1026 | 0.0474 | 0.03035 | Novel:two-stage design |  |
| rs11771693 | 7 | 150050111 | a | g | 0.6743 | 0.169 | 0.0502 | 0.000757 | Novel:two-stage design |  |
| rs10274928 | 7 | 28142088 | a | g | 0.4932 | 0.1644 | 0.0475 | 0.000538 | Novel:two-stage design |  |
| rs10233127 | 7 | 30933453 | a | t | 0.1087 | 0.2638 | 0.0805 | 0.001051 | Novel:two-stage design |  |
| rs6593297 | 7 | 56122058 | a | t | 0.3178 | 0.0982 | 0.0523 | 0.06052 | Novel:two-stage design | CAD |
| rs6963105 | 7 | 75097488 | a | g | 0.4432 | -0.2035 | 0.0531 | 0.000127 | Novel:two-stage design |  |
| rs848445 | 7 | 77572461 | t | c | 0.2821 | -0.2067 | 0.0528 | 9.04E-05 | Novel:two-stage design |  |
| rs17477177 | 7 | 106411858 | t | c | 0.7906 | -0.5642 | 0.0564 | 1.60E-23 | Previously reported | CVD, Smoking |
| rs4728142 | 7 | 128573967 | a | g | 0.4383 | -0.2155 | 0.0467 | 3.91E-06 | Previously reported |  |
| rs13238550 | 7 | 131059056 | a | g | 0.3909 | 0.1695 | 0.0472 | 0.000329 | Previously reported |  |
| rs10224002 | 7 | 151415041 | a | g | 0.7186 | -0.2375 | 0.0525 | 5.99E-06 | Previously reported | Diabetes, Cystatin C levels, Hematocrit, Hemoglobin |
| rs6969780 | 7 | 27159136 | c | g | 0.0961 | 0.3697 | 0.0793 | 3.12E-06 | Previously reported |  |
| rs142449193 | 8 | 102750597 | t | c | 0.0491 | -0.4354 | 0.112 | 0.000102 | Novel:one-stage design |  |
| rs4875958 | 8 | 1721090 | a | g | 0.7099 | 0.2209 | 0.0515 | 1.83E-05 | Novel:one-stage design |  |
| rs2979470 | 8 | 30288272 | t | c | 0.4873 | 0.2114 | 0.046 | 4.25E-06 | Novel:one-stage design |  |
| rs2354862 | 8 | 64501744 | a | c | 0.6441 | 0.2139 | 0.0485 | 1.03E-05 | Novel:one-stage design |  |
| rs13253358 | 8 | 68920135 | t | c | 0.297 | 0.1945 | 0.0504 | 0.000113 | Novel:one-stage design |  |
| rs61040371 | 8 | 8503700 | t | c | 0.6221 | 0.191 | 0.0475 | 5.68E-05 | Novel:one-stage design |  |
| rs62526122 | 8 | 92769569 | a | g | 0.2707 | 0.1739 | 0.0557 | 0.001806 | Novel:one-stage design |  |
| rs1986971 | 8 | 10268736 | a | g | 0.7048 | 0.2632 | 0.051 | 2.49E-07 | Novel:two-stage design |  |
| rs4598218 | 8 | 129483956 | t | c | 0.614 | 0.1523 | 0.048 | 0.001523 | Novel:two-stage design |  |
| rs4129585 | 8 | 143312933 | a | c | 0.4438 | 0.1977 | 0.0467 | 2.30E-05 | Novel:two-stage design |  |
| rs1906672 | 8 | 38130025 | a | g | 0.2275 | 0.2644 | 0.055 | 1.51E-06 | Novel:two-stage design |  |
| rs6996733 | 8 | 60535824 | t | c | 0.8439 | 0.1904 | 0.0647 | 0.003269 | Novel:two-stage design |  |
| rs72688070 | 8 | 81393697 | t | c | 0.1714 | -0.1536 | 0.0621 | 0.01338 | Novel:two-stage design |  |
| rs62491354 | 8 | 9730663 | a | g | 0.1401 | 0.3376 | 0.0663 | 3.59E-07 | Novel:two-stage design |  |
| rs35783704 | 8 | 105966258 | a | g | 0.1092 | -0.5219 | 0.0773 | 1.50E-11 | Previously reported | CVD |
| rs2898290 | 8 | 11433909 | t | c | 0.4835 | 0.3419 | 0.0466 | 2.12E-13 | Previously reported | Smoking |
| rs4841569 | 8 | 11452177 | a | g | 0.4123 | -0.3758 | 0.0511 | 1.94E-13 | Previously reported | Smoking |
| rs6557876 | 8 | 25900675 | t | c | 0.2511 | -0.3667 | 0.0533 | 5.98E-12 | Previously reported |  |
| rs520015 | 9 | 211762 | c | g | 0.5144 | 0.2043 | 0.0456 | 7.60E-06 | Novel:one-stage design |  |
| rs9886665 | 9 | 22942770 | t | c | 0.2721 | 0.1887 | 0.0519 | 0.000277 | Novel:one-stage design | Cancer |
| rs60191654 | 9 | 753648 | a | g | 0.8143 | -0.2311 | 0.0584 | 7.50E-05 | Novel:one-stage design |  |
| rs7023828 | 9 | 128498594 | t | c | 0.423 | -0.2466 | 0.0464 | 1.10E-07 | Novel:two-stage design |  |
| rs1891730 | 9 | 130309028 | t | c | 0.6198 | -0.1749 | 0.0479 | 0.000257 | Novel:two-stage design |  |
| rs184457 | 9 | 131940019 | a | g | 0.2995 | -0.1157 | 0.0498 | 0.02015 | Novel:two-stage design |  |
| rs28558845 | 9 | 4334791 | c | g | 0.1568 | -0.2472 | 0.0652 | 0.00015 | Novel:two-stage design |  |
| rs1332813 | 9 | 9350706 | t | c | 0.3515 | 0.1771 | 0.0472 | 0.000175 | Novel:two-stage design |  |
| rs7045409 | 9 | 95201540 | a | t | 0.3681 | -0.1498 | 0.0473 | 0.001553 | Novel:two-stage design |  |
| rs111245230 | 9 | 113169775 | t | c | 0.9662 | -0.6917 | 0.1299 | 9.99E-08 | Previously reported | CAD |
| rs11592107 | 10 | 122968964 | a | g | 0.3087 | 0.2721 | 0.0495 | 3.93E-08 | Novel:one-stage design |  |
| rs72834453 | 10 | 124235226 | t | g | 0.8742 | -0.2378 | 0.0712 | 0.000839 | Novel:one-stage design |  |
| rs3802517 | 10 | 28233469 | a | t | 0.4668 | 0.188 | 0.0456 | 3.80E-05 | Novel:one-stage design |  |
| rs11187142 | 10 | 94468685 | t | c | 0.1047 | 0.298 | 0.0763 | 9.34E-05 | Novel:one-stage design |  |
| rs11197813 | 10 | 118523933 | a | g | 0.7025 | -0.1612 | 0.0505 | 0.0014 | Novel:two-stage design |  |
| rs7912283 | 10 | 133773019 | a | g | 0.642 | -0.2008 | 0.0505 | 7.02E-05 | Novel:two-stage design |  |
| rs1133400 | 10 | 134459388 | a | g | 0.7954 | -0.307 | 0.0601 | 3.24E-07 | Novel:two-stage design | Hematocrit |
| rs34130368 | 10 | 48411796 | t | g | 0.1197 | -0.1772 | 0.0816 | 0.02998 | Novel:two-stage design | Hematocrit, Hemoglobin |
| rs56352451 | 10 | 5804865 | t | c | 0.1337 | 0.3049 | 0.0672 | 5.69E-06 | Novel:two-stage design |  |
| rs12572586 | 10 | 74751579 | t | c | 0.9383 | -0.4496 | 0.1012 | 8.86E-06 | Novel:two-stage design |  |
| rs112184198 | 10 | 102604514 | a | g | 0.1058 | -0.5331 | 0.0761 | 2.40E-12 | Previously reported |  |
| rs1004467 | 10 | 104594507 | a | g | 0.9028 | 0.8884 | 0.0785 | 1.08E-29 | Previously reported |  |
| rs11191548 | 10 | 104846178 | t | c | 0.9129 | 1.0233 | 0.0818 | 6.19E-36 | Previously reported | Smoking |
| rs4746172 | 10 | 75855842 | t | c | 0.7348 | -0.1017 | 0.0528 | 0.0542 | Previously reported |  |
| rs932764 | 10 | 95895940 | a | g | 0.5561 | -0.3654 | 0.0467 | 4.84E-15 | Previously reported |  |
| rs10766533 | 11 | 19224677 | a | t | 0.7004 | 0.2572 | 0.0515 | 5.85E-07 | Novel:one-stage design |  |
| rs11031051 | 11 | 30355707 | a | c | 0.683 | -0.1902 | 0.0493 | 0.000116 | Novel:two-stage design |  |
| rs190194639 | 11 | 34068037 | t | c | 0.0823 | 0.3274 | 0.0862 | 0.000146 | Novel:two-stage design |  |
| rs1585453 | 11 | 46884713 | a | t | 0.8866 | -0.2449 | 0.0775 | 0.00157 | Novel:two-stage design |  |
| rs4385883 | 11 | 51539339 | a | t | 0.7047 | 0.2189 | 0.0566 | 0.000112 | Novel:two-stage design |  |
| rs4980515 | 11 | 63744609 | t | c | 0.504 | 0.227 | 0.0464 | 1.01E-06 | Novel:two-stage design |  |
| rs67976715 | 11 | 68023742 | c | g | 0.2282 | 0.2708 | 0.0555 | 1.04E-06 | Novel:two-stage design |  |
| rs10743086 | 11 | 8774923 | a | g | 0.2086 | -0.2193 | 0.0567 | 0.000111 | Novel:two-stage design |  |
| rs7129220 | 11 | 10350538 | a | g | 0.1233 | 0.3919 | 0.0724 | 6.28E-08 | Previously reported |  |
| rs1401454 | 11 | 16250183 | t | c | 0.3998 | 0.3365 | 0.0469 | 7.10E-13 | Previously reported |  |
| rs757081 | 11 | 17351683 | c | g | 0.6644 | -0.2958 | 0.0487 | 1.29E-09 | Previously reported | BMI |
| rs5219 | 11 | 17409572 | t | c | 0.3755 | 0.32 | 0.0471 | 1.12E-11 | Previously reported | BMI, Diabetes |
| rs661348 | 11 | 1905292 | t | c | 0.5632 | -0.3417 | 0.0502 | 9.56E-12 | Previously reported | Smoking |
| rs217727 | 11 | 2016908 | a | g | 0.192 | 0.3626 | 0.061 | 2.85E-09 | Previously reported | Smoking, Cancer |
| rs11537751 | 11 | 47587452 | t | c | 0.0521 | 0.3936 | 0.1076 | 0.000256 | Previously reported |  |
| rs11229457 | 11 | 58207203 | t | c | 0.2144 | -0.2886 | 0.0563 | 2.97E-07 | Previously reported |  |
| rs3741378 | 11 | 65408937 | t | c | 0.1328 | -0.4169 | 0.0696 | 2.15E-09 | Previously reported | Smoking, BMI |
| rs7927515 | 11 | 76125330 | a | c | 0.3455 | 0.1705 | 0.0488 | 0.000479 | Previously reported |  |
| rs117206641 | 12 | 133086888 | t | c | 0.1145 | 0.3348 | 0.0783 | 1.88E-05 | Novel:one-stage design | VHD |
| rs28621435 | 12 | 13860990 | a | g | 0.1187 | -0.3138 | 0.0729 | 1.69E-05 | Novel:one-stage design |  |
| rs4143175 | 12 | 67782397 | t | c | 0.239 | 0.3055 | 0.0533 | 9.90E-09 | Novel:one-stage design |  |
| rs5742643 | 12 | 102837863 | t | c | 0.2505 | -0.2603 | 0.0534 | 1.07E-06 | Novel:two-stage design |  |
| rs11112548 | 12 | 105871914 | a | t | 0.9558 | 0.5768 | 0.1203 | 1.64E-06 | Novel:two-stage design |  |
| rs11571376 | 12 | 1059556 | c | g | 0.7011 | -0.1164 | 0.0506 | 0.0215 | Novel:two-stage design |  |
| rs2024385 | 12 | 12888438 | a | t | 0.4186 | -0.243 | 0.0467 | 1.99E-07 | Novel:two-stage design |  |
| rs7976167 | 12 | 24210599 | t | c | 0.6893 | 0.1409 | 0.0489 | 0.003922 | Novel:two-stage design |  |
| rs10437954 | 12 | 58003922 | a | g | 0.9064 | -0.4326 | 0.0832 | 2.01E-07 | Novel:two-stage design |  |
| rs7963801 | 12 | 79685226 | t | c | 0.4129 | -0.2145 | 0.0482 | 8.45E-06 | Novel:two-stage design |  |
| rs10858966 | 12 | 90567026 | c | g | 0.3035 | 0.2024 | 0.05 | 5.12E-05 | Novel:two-stage design |  |
| rs2384550 | 12 | 115352731 | a | g | 0.3457 | -0.2748 | 0.0473 | 6.29E-09 | Previously reported |  |
| rs1126930 | 12 | 49399132 | c | g | 0.0343 | 0.5757 | 0.14 | 3.93E-05 | Previously reported | BMI, HDL, RBC, HbA1c |
| rs73099903 | 12 | 53440779 | t | c | 0.0794 | 0.4218 | 0.0878 | 1.56E-06 | Previously reported |  |
| rs7297416 | 12 | 54443090 | a | c | 0.6867 | 0.2816 | 0.05 | 1.84E-08 | Previously reported |  |
| rs2681492 | 12 | 90013089 | t | c | 0.8344 | 0.7729 | 0.0615 | 3.26E-36 | Previously reported | CAD, CVD, AF |
| rs17249754 | 12 | 90060586 | a | g | 0.1637 | -0.8015 | 0.0619 | 2.16E-38 | Previously reported | AF, Smoking |
| rs2480171 | 13 | 21559858 | t | c | 0.1324 | 0.2057 | 0.0693 | 0.002978 | Novel:one-stage design |  |
| rs1331012 | 13 | 27115424 | t | g | 0.269 | 0.1514 | 0.051 | 0.002962 | Novel:one-stage design |  |
| rs4274337 | 13 | 41967193 | a | g | 0.177 | -0.33 | 0.0612 | 6.93E-08 | Novel:one-stage design |  |
| rs75961402 | 13 | 56398286 | a | g | 0.1516 | 0.2759 | 0.0635 | 1.40E-05 | Novel:one-stage design |  |
| rs606950 | 13 | 22298923 | a | g | 0.6176 | 0.1755 | 0.047 | 0.000186 | Novel:two-stage design |  |
| rs9532243 | 13 | 32191408 | a | c | 0.4797 | 0.2485 | 0.0452 | 3.89E-08 | Novel:two-stage design |  |
| rs73187288 | 13 | 42738672 | a | c | 0.8935 | -0.2492 | 0.0738 | 0.000731 | Novel:two-stage design |  |
| rs912434 | 13 | 47189928 | t | g | 0.7628 | 0.2107 | 0.0531 | 7.30E-05 | Novel:two-stage design |  |
| rs9526707 | 13 | 51489186 | a | g | 0.3166 | -0.2364 | 0.0492 | 1.56E-06 | Novel:two-stage design |  |
| rs78474310 | 13 | 73826901 | a | g | 0.9566 | -0.4412 | 0.1138 | 0.000106 | Novel:two-stage design |  |
| rs7988232 | 13 | 79808655 | a | g | 0.4146 | 0.1378 | 0.0463 | 0.002917 | Novel:two-stage design |  |
| rs3011549 | 13 | 113634937 | a | c | 0.2888 | 0.226 | 0.0539 | 2.78E-05 | Previously reported |  |
| rs63418562 | 13 | 30146201 | t | c | 0.7462 | -0.3846 | 0.0529 | 3.74E-13 | Previously reported |  |
| rs34983854 | 14 | 39858442 | a | g | 0.6064 | -0.2259 | 0.0463 | 1.05E-06 | Novel:one-stage design |  |
| rs8014182 | 14 | 103859962 | t | c | 0.1388 | -0.3218 | 0.0655 | 8.80E-07 | Novel:two-stage design |  |
| rs17115145 | 14 | 30122409 | t | c | 0.3909 | 0.1853 | 0.0462 | 6.08E-05 | Novel:two-stage design |  |
| rs72683923 | 14 | 50735947 | t | c | 0.9767 | 0.7823 | 0.1705 | 4.45E-06 | Novel:two-stage design | AF |
| rs11623535 | 14 | 72462381 | a | g | 0.7393 | 0.1623 | 0.0513 | 0.001552 | Novel:two-stage design |  |
| rs11159091 | 14 | 75074316 | a | g | 0.4654 | 0.1973 | 0.046 | 1.78E-05 | Novel:two-stage design |  |
| rs9888615 | 14 | 53377540 | t | c | 0.2936 | -0.2356 | 0.0499 | 2.32E-06 | Previously reported |  |
| rs8016306 | 14 | 63928546 | a | g | 0.7931 | 0.1339 | 0.0554 | 0.01569 | Previously reported |  |
| rs4965529 | 15 | 100145224 | a | c | 0.1657 | -0.2802 | 0.0622 | 6.60E-06 | Novel:two-stage design |  |
| rs11634028 | 15 | 76276150 | a | t | 0.205 | 0.2356 | 0.059 | 6.49E-05 | Novel:two-stage design |  |
| rs3743157 | 15 | 85680532 | a | c | 0.1651 | 0.2069 | 0.0615 | 0.000766 | Novel:two-stage design |  |
| rs11632436 | 15 | 86295286 | c | g | 0.5045 | 0.1907 | 0.0458 | 3.07E-05 | Novel:two-stage design |  |
| rs35199222 | 15 | 81013037 | a | g | 0.4398 | 0.2436 | 0.0466 | 1.75E-07 | Previously reported |  |
| rs2759308 | 15 | 81016227 | a | g | 0.4758 | 0.2592 | 0.046 | 1.79E-08 | Previously reported |  |
| rs2379829 | 16 | 3538873 | c | g | 0.728 | -0.2143 | 0.0521 | 3.84E-05 | Novel:one-stage design |  |
| rs34941092 | 16 | 50550137 | a | g | 0.1491 | -0.302 | 0.0651 | 3.53E-06 | Novel:one-stage design |  |
| rs1012089 | 16 | 74171973 | c | g | 0.4758 | -0.1354 | 0.0456 | 0.002974 | Novel:one-stage design |  |
| rs3851018 | 16 | 86437811 | c | g | 0.5676 | 0.2224 | 0.0473 | 2.60E-06 | Novel:one-stage design |  |
| rs6540125 | 16 | 87993889 | t | g | 0.3501 | 0.1864 | 0.0475 | 8.75E-05 | Novel:one-stage design |  |
| rs35450617 | 16 | 6889675 | t | g | 0.6958 | -0.1542 | 0.051 | 0.002489 | Novel:two-stage design |  |
| rs7187540 | 16 | 85318302 | a | c | 0.3245 | -0.193 | 0.0563 | 0.000606 | Novel:two-stage design |  |
| rs9899540 | 17 | 30777924 | a | t | 0.4126 | 0.1809 | 0.0487 | 0.0002 | Novel:one-stage design |  |
| rs112260610 | 17 | 64252393 | t | c | 0.1353 | 0.3389 | 0.0669 | 4.11E-07 | Novel:one-stage design |  |
| rs4925159 | 17 | 18185510 | a | g | 0.4192 | 0.2134 | 0.0464 | 4.23E-06 | Novel:two-stage design |  |
| rs1551355 | 17 | 30032420 | t | c | 0.2369 | 0.1842 | 0.0538 | 0.000621 | Novel:two-stage design |  |
| rs34430710 | 17 | 56876627 | a | t | 0.6753 | -0.2151 | 0.0487 | 9.87E-06 | Novel:two-stage design |  |
| rs1036902 | 17 | 58950791 | t | c | 0.8404 | -0.2107 | 0.0634 | 0.000888 | Novel:two-stage design |  |
| rs112280096 | 17 | 79367409 | a | c | 0.37 | -0.0932 | 0.0561 | 0.09643 | Novel:two-stage design |  |
| rs12946454 | 17 | 43208121 | a | t | 0.739 | -0.3193 | 0.0518 | 7.30E-10 | Previously reported |  |
| rs7406910 | 17 | 46688256 | t | c | 0.0893 | -0.4877 | 0.0812 | 1.93E-09 | Previously reported | AF |
| rs8068318 | 17 | 59483766 | t | c | 0.7271 | 0.4318 | 0.0536 | 8.20E-16 | Previously reported | AF, Creatinine levels |
| rs2240736 | 17 | 59485393 | t | c | 0.7328 | 0.4265 | 0.0525 | 4.49E-16 | Previously reported | AF |
| rs1154214 | 18 | 24546824 | t | g | 0.3963 | -0.2163 | 0.046 | 2.57E-06 | Novel:one-stage design |  |
| rs6567160 | 18 | 57829135 | t | c | 0.7644 | 0.1618 | 0.0541 | 0.002765 | Novel:one-stage design | BMI, Smoking, Diabetes |
| rs10460108 | 18 | 73034151 | a | g | 0.4819 | 0.2039 | 0.0452 | 6.40E-06 | Novel:one-stage design |  |
| rs11876341 | 18 | 48799991 | a | g | 0.6949 | -0.2167 | 0.0518 | 2.89E-05 | Novel:two-stage design |  |
| rs10048404 | 18 | 54578482 | t | c | 0.3741 | -0.2123 | 0.049 | 1.46E-05 | Novel:two-stage design | Diabetes, AF, |
| rs12454712 | 18 | 60845884 | t | c | 0.6224 | 0.1891 | 0.0537 | 0.000429 | Novel:two-stage design | BMI, Smoking, Diabetes |
| rs34413141 | 18 | 777282 | a | t | 0.1796 | -0.337 | 0.0599 | 1.83E-08 | Novel:two-stage design |  |
| rs12958173 | 18 | 42141977 | a | c | 0.3 | 0.3518 | 0.0495 | 1.21E-12 | Previously reported |  |
| rs7256564 | 19 | 33889593 | a | g | 0.3133 | 0.2039 | 0.0487 | 2.87E-05 | Novel:one-stage design | TG |
| rs73046792 | 19 | 49605705 | a | g | 0.1513 | -0.2413 | 0.069 | 0.000474 | Novel:one-stage design | AF |
| rs2613765 | 19 | 5066330 | a | g | 0.4768 | -0.1874 | 0.0455 | 3.85E-05 | Novel:two-stage design | AF |
| rs138877676 | 19 | 50935809 | t | g | 0.0211 | -0.5482 | 0.2033 | 0.006999 | Novel:two-stage design |  |
| rs17638167 | 19 | 11584818 | t | c | 0.047 | -0.5228 | 0.1095 | 1.81E-06 | Previously reported |  |
| rs8105753 | 19 | 31927547 | a | c | 0.6255 | 0.1895 | 0.0487 | 9.88E-05 | Previously reported |  |
| rs4247374 | 19 | 7252756 | t | c | 0.1355 | -0.5063 | 0.0753 | 1.76E-11 | Previously reported | Smoking |
| rs1764975 | 20 | 4101290 | a | t | 0.7894 | 0.2759 | 0.058 | 1.99E-06 | Novel:one-stage design |  |
| rs6021247 | 20 | 50108980 | a | g | 0.5289 | 0.1623 | 0.0453 | 0.000338 | Novel:two-stage design | CVD, AF |
| rs6031435 | 20 | 42797358 | a | g | 0.5388 | -0.2268 | 0.0456 | 6.72E-07 | Previously reported | AF |
| rs11701033 | 21 | 33788341 | c | g | 0.8169 | -0.2465 | 0.0592 | 3.18E-05 | Previously reported |  |
| rs9608690 | 22 | 28921347 | a | g | 0.0678 | -0.308 | 0.0912 | 0.000733 | Novel:one-stage design |  |
| rs28578714 | 22 | 50727921 | t | c | 0.6045 | 0.2346 | 0.0538 | 1.28E-05 | Novel:two-stage design |  |
| * Candidate SNPs selected from Evangelou et al. ^11^ † Regression coefficient and corresponding standard error derived from International Consortium for Blood Pressure Genome-Wide Association Studies (ICBP). ^10^  CRP: C-Reactive Protein, LDL: low-density lipoprotein, TG: Triglycerides, AF: atrial fibrillation, CKD: Chronic kidney disease, WBC: white blood cells, RBC: red blood cells, BMI: body mass index, CAD: coronary artery diseases, HDL: high-density lipoprotein, CVD: cardiovascular diseases, VLDL: | | | | | | | | | |  |

| Table S3. Sensitivity analysis adjusting the one-sample Mendelian randomization for comorbid cardiometabolic conditions. | | |
| --- | --- | --- |
| **Analysis without adjustment** | |  |
| Outcomes | OR (95% CIs) |  |
| Venous thromboembolism | 0.69 (0.57 to 0.83) |  |
| Pulmonary embolism | 0.71 (0.55 to 0.92) |  |
| Deep vein thrombosis | 0.72 (0.56 to 0.92) |  |
| **Analysis adjusted for comorbid cardiometabolic diseases *** | | |
| Venous thromboembolism | 0.63 (0.52 to 0.76) |  |
| Pulmonary embolism | 0.65 (0.50 to 0.84) |  |
| Deep vein thrombosis | 0.66 (0.51 to 0.85) |  |
| OR: odds ratio, CIs, confidence intervals  * Diabetes, heart failure, coronary heart disease, atrial fibrillation, stroke, valvular heart diseases | | |

| Table S4. Sensitivity analysis excluding participants with a history of blood pressure treatment use in one-sample Mendelian randomization. | | | |
| --- | --- | --- | --- |
| Outcomes | Weighted GRS | | Unweighted GRS |
|  | OR (95% CIs) |  | OR (95% CIs) |
| Venous thromboembolism | 0.71 (0.57 to 0.88) |  | 0.75 (0.61 to 0.94) |
| Pulmonary embolism | 0.76 (0.57 to 1.01) |  | 0.83 (0.62 to 1.11) |
| Deep vein thrombosis | 0.75 (0.57 to 0.99) |  | 0.79 (0.59 to 1.04) |
| OR: odds ratio, CIs, confidence intervals, GRS: genetic risk score | | | |

| Table S5. Sensitivity analysis excluding participants with at least one relative to other participants in one-sample Mendelian randomization. | | |
| --- | --- | --- |
| Outcomes | OR (95% CIs) |  |
| Venous thromboembolism | 0.71 (0.57 to 0.88) |  |
| Pulmonary embolism | 0.76 (0.57 to 1.01) |  |
| Deep vein thrombosis | 0.75 (0.57 to 0.99) |  |
| OR: odds ratio, CIs, confidence intervals | | |

##

## Figure S1. The hazard ratio for venous thromboembolism per 20 mmHg increase in systolic blood pressure with progressive adjustment.


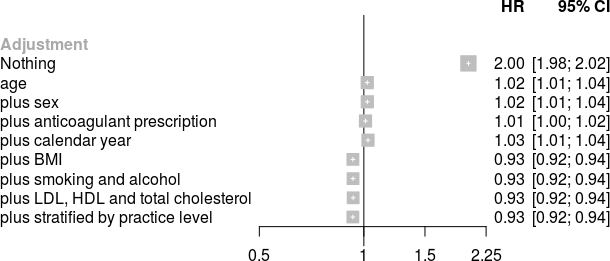


## Figure S2. Sensitivity analysis of the association between systolic blood pressure and the risk of venous thromboembolism stratified based on objective criteria for outcome definition.

**
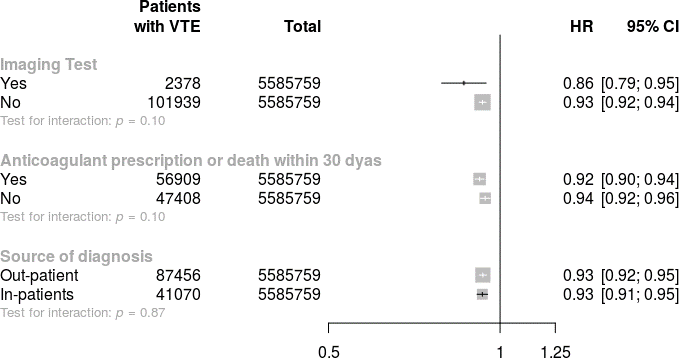
**

## Figure S3. The distribution of genetic risk score and measured systolic blood pressure in the one-sample Mendelian randomization.


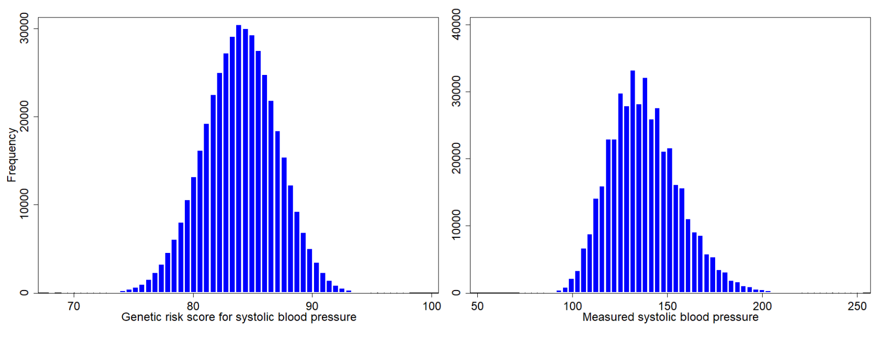


| Characteristics of participants in one-sample Mendelian randomization analysis according to quartile of genetic risk score for systolic blood pressure. | | | | |
| --- | --- | --- | --- | --- |
|  | **Quartiles of genetic risk score** | | |  |
| **Characteristics** | ≤ 82 | 83-84 | 85-86 | > 86 |
| Measured SBP [mmHg], mean (SD) | 135.4 (18.0) | 137.4 (18.4) | 138.9 (18.6) | 140.8 (18.9) |
| Age, mean (SD) | 56.8 (7.9) | 56.8 (8.0) | 56.8 (7.9) | 56.8 (8.0) |
| Sex, n (%) |  |  |  |  |
| Women | 58392 (54.1) | 58150 (53.9) | 584241 (54.1) | 58413 (54.1) |
| Men | 49596 (45.9) | 49834 (46.1) | 49568 (45.9) | 49573 (45.9) |
| Body mass index [Kg/m^2^], mean (SD) | 27.4 (4.7) | 27.4 (4.7) | 27.4 (4.7) | 27.4 (4.7) |
| Total cholestrol [mmol/L], mean (SD) | 5.7 (1.1) | 5.7 (1.1) | 5.7 (1.1) | 5.6 (1.1) |
| Blood glocose [mmol/L],, mean (SD) | 5.1 (1.1) | 5.1 (1.2) | 5.1 (1.2) | 5.1 (1.2) |
| Triglyceride [mmol/L], mean (SD) | 1.7 (1.02) | 1.7 (1.03) | 1.7 (1.02) | 1.7 (1.01) |
| SBP: systolic blood pressure, SD: standard deviation | | | |  |
|  |  |  |  |  |

## Figure S4. Positive control analysis to test the validity of the instrumental variable.


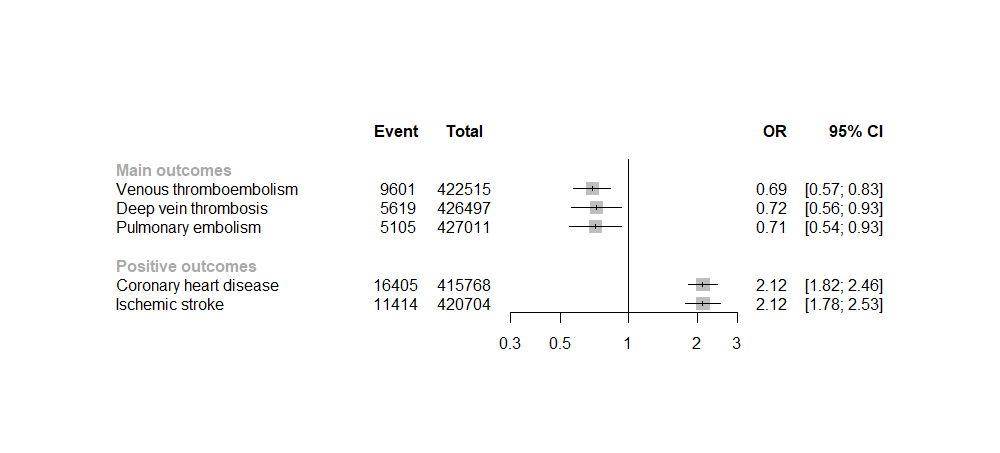


## Figure S5. Sensitivity analysis using an unweighted polygenic risk score to check the robustness of the weighting approach


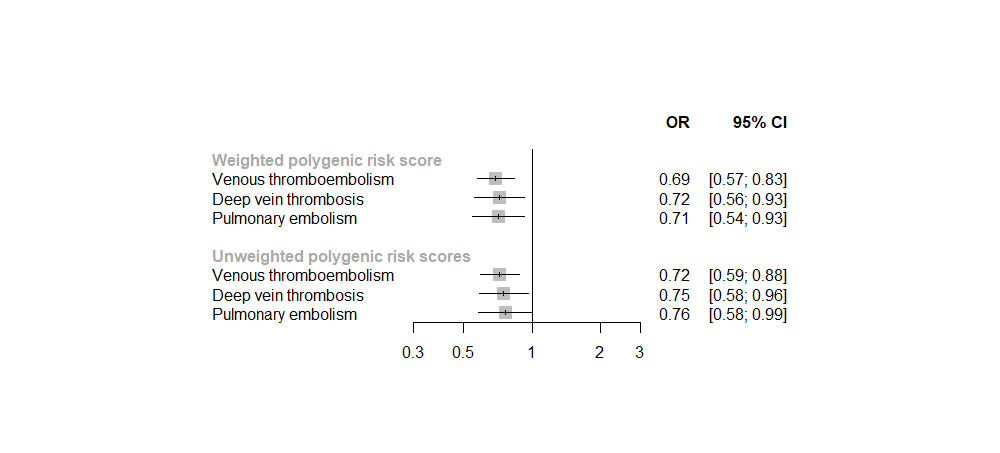


## Figure S6. Sensitivity analysis to assess the effect of adjusting for potential confounders on the main estimations.

Fully adjusted model: the model further adjusted for body mass index, alcohol intake frequency, smoking status, total cholesterol, low-density lipoprotein (LDL), high-density lipoprotein (HDL) and blood pressure-lowering medications use. OR: odds ratio


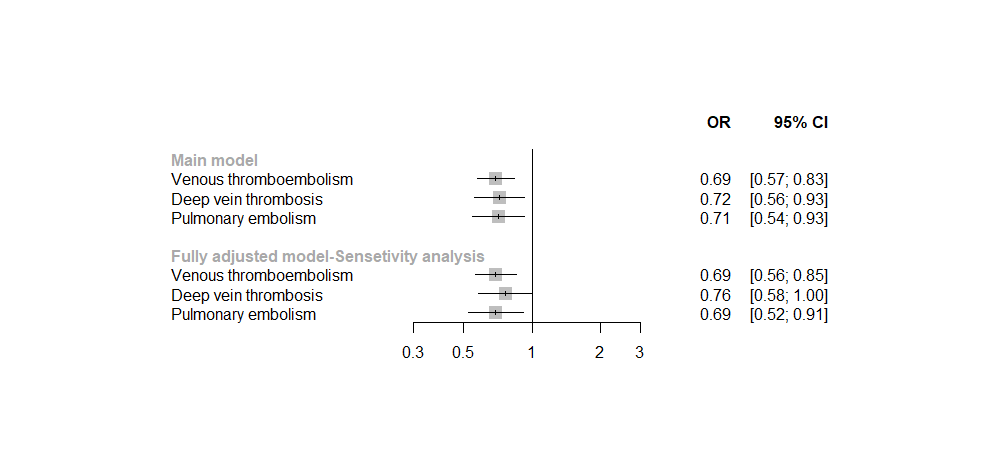


## Figure S7. Scatter plot of 276 variants associated with systolic blood pressure and their effect on venous thromboembolism.


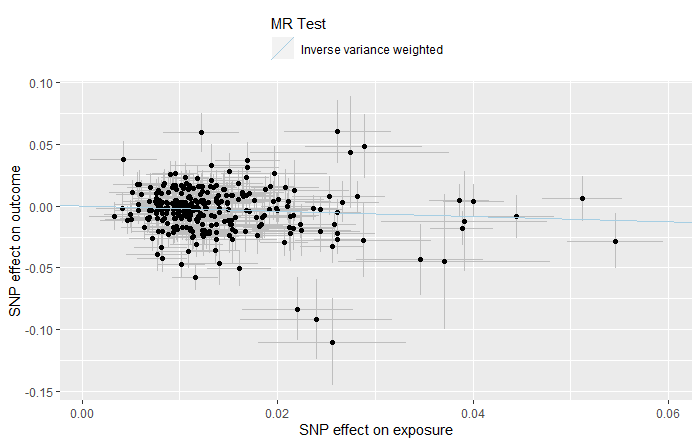


#

## Figure S8. Funnel plot of 276 variants, showing each variant causal estimate against instrument strength.


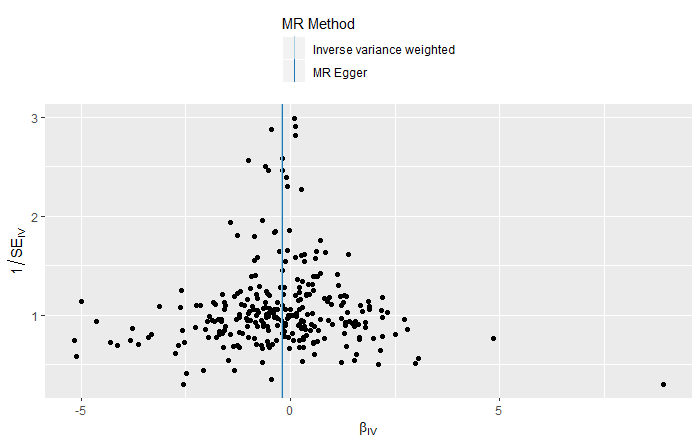


## Figure S9. Sensitivity analysis methods of two-sample Mendelian randomization to assess the causal association between systolic blood pressure and risk of venous thromboembolism.

All variants: Analysis included all the 276 genetic variants

Excluding pleiotropic variants: sensitivity analysis excluding 61 variants associated with any type of well-known cardiovascular risk factors or diseases (Supplementary Table S2). OR: odds ratio, CI: confidence interval

For the second part of analysis, first, the individual association of each variant with other risk factors or cardiovascular diseases has been checked using the NHGRI-EBI Catalog of published genome-wide association studies (https://www.ebi.ac.uk/gwas/home). We then conducted a sensitivity analysis excluding 61 variants associated with any type of well-known cardiovascular risk factors or diseases (Supplementary Table S2).


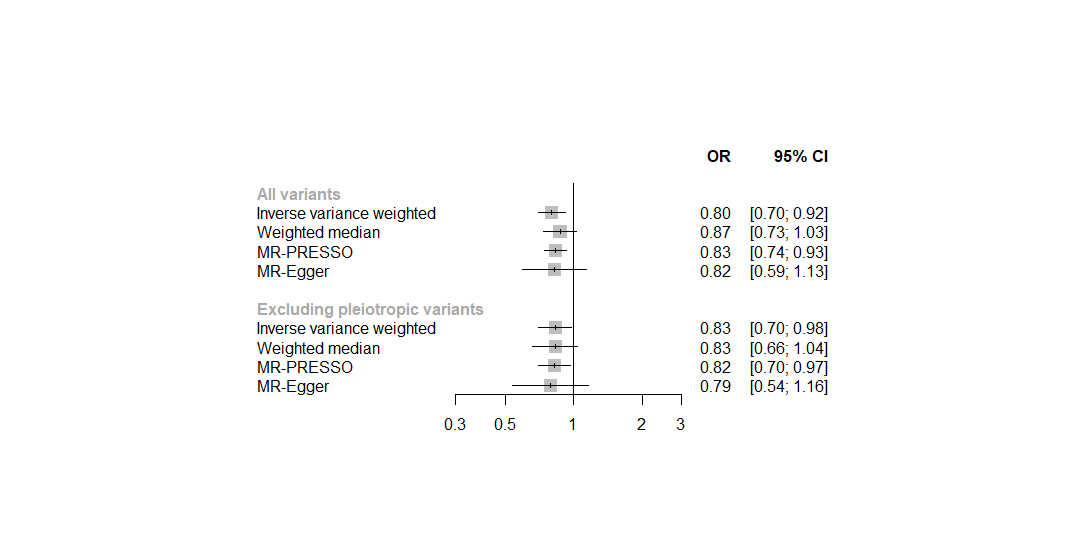


## References

1. Herrett E, Gallagher AM, Bhaskaran K, Forbes H, Mathur R, Staa T van, Smeeth L. Data Resource Profile: Clinical Practice Research Datalink (CPRD). *International Journal of Epidemiology* 2015;**44**:827–836.

2. García Rodríguez LA, Pérez Gutthann S. Use of the UK General Practice Research Database for pharmacoepidemiology. *British journal of clinical pharmacology* 1998;**45**:419–425.

3. Herrett E, Thomas SL, Schoonen WM, Smeeth L, Hall AJ. Validation and validity of diagnoses in the General Practice Research Database: A systematic review. *British Journal of Clinical Pharmacology* 2010;**69**:4–14.

4. Khan NF, Harrison SE, Rose PW. Validity of diagnostic coding within the General Practice Research Database: A systematic review. *British Journal of General Practice* 2010;**60**:199–206.

5. Easton DF, Peto J, Babiker AG. Floating absolute risk: an alternative to relative risk in survival and case-control analysis avoiding an arbitrary reference group. *Statistics in medicine* 1991;**10**:1025–1035.

6. Easton DF, Peto J, Babiker AGAG. Floating absolute risk: An alternative to relative risk in survival and case-control analysis avoiding an arbitrary reference group. *Statistics in Medicine* John Wiley & Sons, Ltd; 1991;**10**:1025–1035.

7. Hutcheon JA, Chiolero A, Hanley JA. Random measurement error and regression dilution bias. *BMJ (Clinical research ed)* British Medical Journal Publishing Group; 2010;**340**:c2289.

8. Evangelou E, Warren HR, Mosen-Ansorena D, Mifsud B, Pazoki R, Gao H, Ntritsos G, Dimou N, Cabrera CP, Karaman I, Ng FL, Evangelou M, Witkowska K, Tzanis E, Hellwege JN, Giri A, Edwards DRV, Sun Y V., Cho K, Gaziano JM, Wilson PWF, Tsao PS, Kovesdy CP, Esko T, Magi R, Milani L, Almgren P, Boutin T, Debette S, Ding J, et al. Genetic analysis of over 1 million people identifies 535 new loci associated with blood pressure traits. *Nature Genetics* Cold Spring Harbor Laboratory; 2018;

9. Burgess S, Davies NM, Thompson SG. Bias due to participant overlap in two-sample Mendelian randomization. *Genetic Epidemiology* Wiley-Liss Inc.; 2016;**40**:597–608.

10. Ehret GB, Munroe PB, Rice KM, Bochud M, Johnson AD, Chasman DI, Smith A V., Tobin MD, Verwoert GC, Hwang SJ, Pihur V, Vollenweider P, O’Reilly PF, Amin N, Bragg-Gresham JL, Teumer A, Glazer NL, Launer L, Zhao JH, Aulchenko Y, Heath S, Sober S, Parsa A, Luan J, Arora P, Dehghan A, Zhang F, Lucas G, Hicks AA, Jackson AU, et al. Genetic variants in novel pathways influence blood pressure and cardiovascular disease risk. *Nature* Nature; 2011;**478**:103–109.

11. Evangelou E, Warren HR, Mosen-Ansorena D, Mifsud B, Pazoki R, Gao H, Ntritsos G, Dimou N, Cabrera CP, Karaman I, Ng FL, Evangelou M, Witkowska K, Tzanis E, Hellwege JN, Giri A, Velez Edwards DR, Sun Y V., Cho K, Gaziano JM, Wilson PWF, Tsao PS, Kovesdy CP, Esko T, Mägi R, Milani L, Almgren P, Boutin T, Debette S, Ding J, et al. Genetic analysis of over 1 million people identifies 535 new loci associated with blood pressure traits. *Nature Genetics* Nature Publishing Group; 2018;**50**:1412–1425.
